# A Review of Landfill Microbiology and Ecology: A Call for Modernization With ‘Next Generation’ Technology

**DOI:** 10.3389/fmicb.2020.01127

**Published:** 2020-06-03

**Authors:** D’Arcy R. Meyer-Dombard, Jean E. Bogner, Judy Malas

**Affiliations:** Department of Earth and Environmental Sciences, University of Illinois at Chicago, Chicago, IL, United States

**Keywords:** municipal solid waste, landfills, methane, environmental microbiology, engineered ecosystems

## Abstract

Engineered and monitored sanitary landfills have been widespread in the United States since the passage of the Clean Water Act (1972) with additional controls under RCRA Subtitle D (1991) and the Clean Air Act Amendments (1996). Concurrently, many common perceptions regarding landfill biogeochemical and microbiological processes and estimated rates of gas production also date from 2 to 4 decades ago. Herein, we summarize the recent application of modern microbiological tools as well as recent metadata analysis using California, USEPA and international data to outline an evolving view of landfill biogeochemical/microbiological processes and rates. We focus on United States landfills because these are uniformly subject to stringent national and state requirements for design, operations, monitoring, and reporting. From a microbiological perspective, because anoxic conditions and methanogenesis are rapidly established after daily burial of waste and application of cover soil, the >1000 United States landfills with thicknesses up to >100 m form a large ubiquitous group of dispersed ‘dark’ ecosystems dominated by anaerobic microbial decomposition pathways for food, garden waste, and paper substrates. We review past findings of landfill ecosystem processes, and reflect on the potential impact that application of modern sequencing technologies (e.g., high throughput platforms) could have on this area of research. Moreover, due to the ever evolving composition of landfilled waste reflecting transient societal practices, we also consider unusual microbial processes known or suspected to occur in landfill settings, and posit areas of research that will be needed in coming decades. With growing concerns about greenhouse gas emissions and controls, the increase of chemicals of emerging concern in the waste stream, and the potential resource that waste streams represent, application of modernized molecular and microbiological methods to landfill ecosystem research is of paramount importance.

## Introduction

### What Is a ‘Landfill?’

‘Landfilling’ varies depending on geopolitical region and encompasses a wide range of regulated and unregulated practices. Landfilled solid waste may or may not be sorted, may or may not include non-municipal components (industrial, commercial, construction, forestry, mining), and may or may not be disposed in an engineered or monitored setting. Individual landfills include diverse geochemical settings with complex microbial ecosystems ranging from deeply buried anaerobic methanogenic systems to near surface aerobic systems. All landfills evolve geochemically and microbiologically with changing environmental conditions which may be zoned or mixed, at various spatial and temporal scales, depending on the site.

**FIGURE 1:**
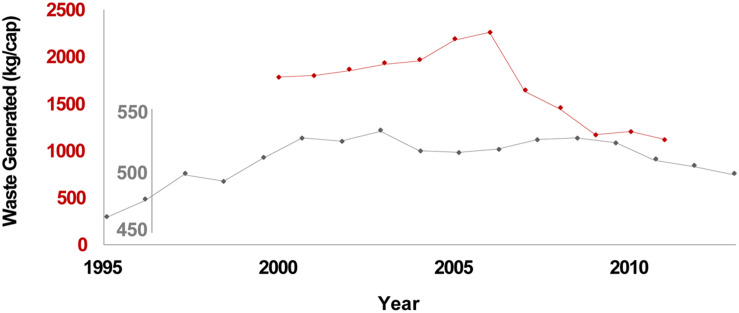
California waste generation (red) and EU27 municipal waste generation (gray). Sources for CA and EU27 data are the California Department of Resources Recovery and Recycling (CalRecycle), and Eurostat, respectively.

Broadly, landfills are large scale landscape features consisting of millions of Mg of waste composed of both anthropogenic and natural organic matter, inorganic constituents, and buried local soils. Landfills are thus relevant to: (1) human induced alterations to the microbiology and microbial ecology of near surface soils, recently reviewed on a global basis by Crowther et al. (2019); (2) longer term temporal considerations relevant to an evolving literature on archeological waste spanning the last several thousand years of the Anthropocene (e.g., Stephens et al., 2019, provide an overview of urban and agricultural centers from 10,000 BP to the present); and (3) very long term temporal considerations regarding organic C cycling via type III kerogen pathways. Indeed, landfilled waste subjected to elevated heat and pressure with deeper burial over very long geologic timeframes may eventually evolve via kerogen pathways for terrestrial organic C to precursors of humic coals (e.g., Tissot and Welte, 1984). Further, solid waste generation rates are a function of both population and prosperity; where direct data are lacking, various economic indicators have been used as prosperity surrogates (Bogner and Matthews, 1999; Bogner et al., 2007). Waste generation rates track economic conditions; for example, Figure 1 illustrates the 2008–2009 global economic downturn via reduced rates of per capita waste generation in California and the EU.

**FIGURE 2:**
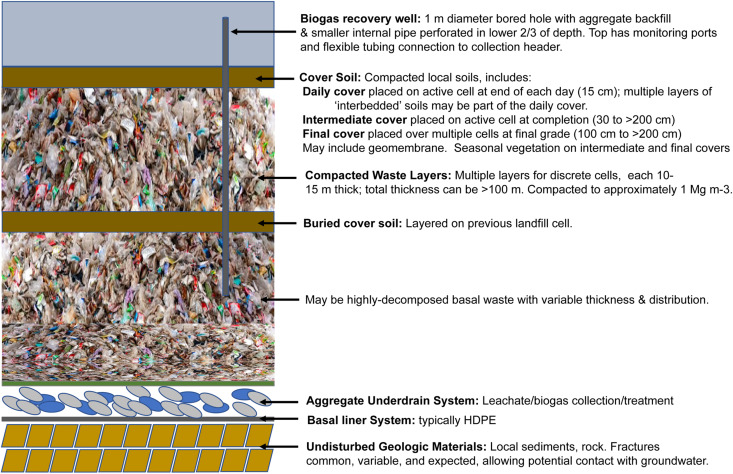
Typical construction for United States landfills, in cross-section.

In highly developed countries with widespread landfilling practices such as the United States, there are stringent regulatory and monitoring requirements for the burial and containment of solid waste: a ‘sanitary landfill’ is an engineered facility with cell construction; bottom and side liners; an underdrain system to intercept liquids (leachate) for removal and treatment; engineered recovery/utilization of methane from anaerobic waste decomposition; placement of daily, intermediate, and final soil cover materials, and decades of monitoring during filling and after closure (Figure 2). In addition to containment, other operational designs that may be applied to engineered landfills include bioreactor designs for accelerated anaerobic decomposition (e.g., Reinhart et al., 2002), designs for accelerated aerobic stabilization (e.g., Ritzkowski and Stegmann, 2012), and semi-aerobic designs which have been implemented in Japan for many years (e.g., Hanashima et al., 1982). In contrast, in many developing countries, landfills consist of large open aerobic dumpsites containing millions of Mg of unsorted solid waste, often with associated scavenging activities, minimal management and associated detrimental impacts on surrounding soil, water, air, and human health (Zyoud et al., 2015; Zhou et al., 2017; Donevska et al., 2018; Ojuri et al., 2018). Management practices in many European countries vary widely (reviewed by Castillo-Giménez et al., 2019). Diaz et al. (1996) summarized large scale surveys of diverse waste composition and landfill practices in economically developing countries. Medina (2007) discusses the economic and societal implications of large scale diversions of putrescible and non-putrescible waste in developing countries prior to landfill disposal, estimating that perhaps 2% of urban populations engage in scavenging activities for their livelihood. Although these activities contribute significantly to urban employment and markets at several economic levels, improved recognition, quantification, and upgraded practices are needed. For historical context, Strasser (1999) traces the evolution of United States waste generation, recycling, and management practices in the United States from the 18th to early 20th century.

Landfills discussed in this review will primarily focus on lined, engineered landfills with an underdrain system for removal of liquids (leachate), biogas recovery concurrent with landfill operations, diversion of surface drainage, and installation of compacted daily, intermediate, and final covers. As appropriate, we will include discussion of less engineered/controlled or monitored systems. We start with a summary of landfill geochemistry to provide background regarding pertinent habitats associated with landfill microbial ecosystems. It should be noted that microbial processes in landfill settings have been studied for more than a half century, relying on geochemical and isotopic approaches, field scale landfill test cells, pilot and full scale anaerobic digesters, site specific monitoring data, use of laboratory microcosms, and the application of older field and laboratory incubation/extraction techniques such as microbial biomass/activity assays and most probable number approaches. Early work was summarized in Halvadakis et al. (1983), a comprehensive literature review undertaken prior to the Mountain View, CA, landfill test cell project to investigate accelerated landfill decomposition at field scale (e.g., see Farquhar and Rovers, 1973, EMCON, 1980, Barlaz et al., 1989a, 1987; Gendebien et al., 1992). In this review, we briefly cover some of the earliest work on landfill microbiology before summarizing what was learned during the first ‘molecular microbiology revolution’ as Sanger-based sequencing methods were applied to both experimental and field samples. Finally, we consider the sparse literature on landfill microbial ecology that makes use of modern, high throughput sequencing methodologies and comment on advances that could be made in future applications of these and developing technologies, as our concepts of the landfill ecosystem evolves.

### Typical Landfill Structure in the United States

Landfills form massive mounds which can be 50+ m high or valley fills >100 m deep. Site design and operations must conform to USEPA Subtitle D of RCCA, Clean Air Act, and state/local engineering standards (Figure 2), including:

(a) A bottom liner to collect downward percolating landfill liquids (leachate) in an aggregate layer above the bottom liner. Leachate is subsequently removed and treated.
(b) The application of three major types of cover materials: *daily*, placed on the active cell at the end of the working day; *intermediate*, placed on top of a given cell at completion; and *final*, placed on top of a group of cells at final grade.
(c) Mandatory installation of biogas collection and recovery via either vertical wells or horizontal collectors, typically when a site has >2 million Mg waste in place. Where economically feasible, biogas utilization also occurs (currently >600 United States sites^1^, most commonly for onsite electrical generation).

With regard to landfill practices, it should be noted that landfill bioreactor designs and operational strategies for more rapid waste decomposition under elevated moisture conditions, typically with leachate recycle, have been studied since at least the early 1980s (i.e., Pohland, 1980; Reinhart and Townsend, 1997; Barlaz et al., 2010). However, adoption has been hindered by a lack of (a) standardized design and operational strategies with predictable capital and operating costs; (b) standardized national regulatory permitting for full scale operations (as opposed to research trials); and (c) comprehensive field validation demonstrating improved biodegradation performance at a large number of diverse sites.

### Municipal Solid Waste (MSW) Composition

A 2012 compilation by the World Bank estimated approximately 1.3 Gt solid waste were being generated by three billion inhabitants of global cities, forecasting an increase to 2.2 Gt generated by 5.6 billion inhabitants by 2015 (Hoornweg and Bhada-Tata, 2012). However, national statistics for the mass and composition of landfilled waste for many developed and developing countries are highly imprecise and will not be addressed in detail in this review. For the EU, Eurostat realistically tracks more than a dozen solid waste streams (i.e., industrial, agricultural, forestry, mining), only one of which is municipal waste routinely managed by urban systems^2^. For the United States, a well-known discrepancy exists between USEPA annual estimates for landfilled solid waste using a material flow model vs. approximately double that quantity based on annual landfilling summaries reported by the various states (Bogner et al., 2007; Van Haaren et al., 2010). More recently, Powell et al. (2016) also supported a higher estimate for the United States by summing the total annual mass of landfilled waste in 2011–2015 reported by landfill owners/operators to the USEPA Greenhouse Gas Reporting Program (GHGRP). Importantly, landfills also store significant quantities of buried organic C (Bogner, 1992; Barlaz, 1998), dominated by lignicellulosic materials. The measured or assumed bulk organic carbon content of landfilled United States waste has historically varied over a relatively small range, roughly 15–25%, with recent literature favoring the lower end of this range (Barlaz, 1998; De la Cruz et al., 2013). Major biodegradable constituents include cellulosics (paper/paper products, plant debris in food and garden waste), as well as fats and proteins in food waste. Worldwide, the composition of landfilled MSW can be highly variable (Asnani, 2006; Krishnamurthi and Chakrabarti, 2013), however, waste data from cities and countries that do not provide systemic waste management practices at the municipal level should be examined with caution. In the United States, MSW landfills may also receive various types of non-hazardous industrial waste, construction and demolition debris, biosolids, and other constituents which are typically itemized at individual entrance weighbridges.

## Landfill Heterogeneity, Chemistry, and Generalized Microbial Processes

**FIGURE 3:**
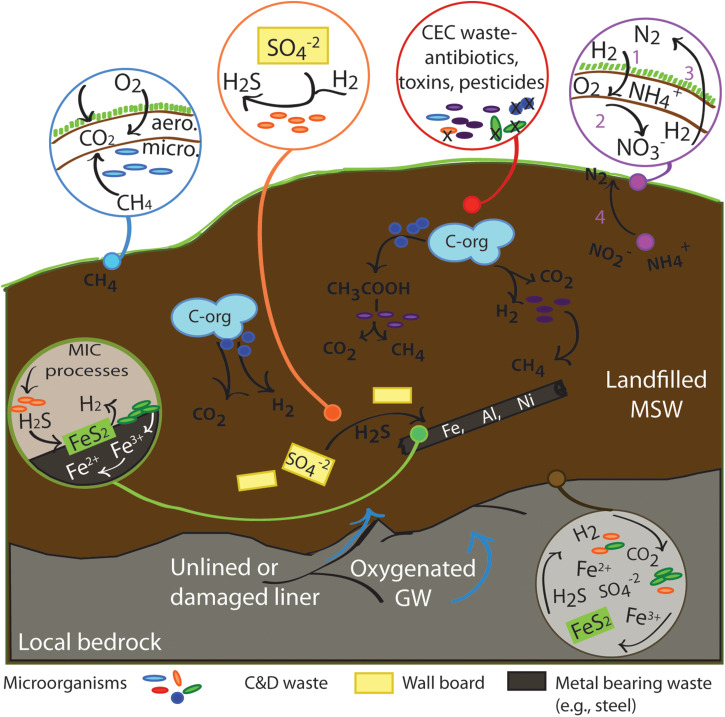
Generalized landfill ecosystem processes, focusing on microbial metabolic functions. Breakdown of organic carbon followed by methanogenesis is represented in the mid-figure. Circular callouts zoom in on specific processes mentioned in the text. Blue callout: Surface and near surface soil processes of methanogenesis and methanotrophy - linked to reactions 1–9 in Table 1. Red callout: Highlighting potential impact of CECs added to landfill ecosystems. CECs may either boost or suppress microbial metabolic processes, or kill specific groups of organisms. Purple callout: Nitrogen cycle processes. Numbered steps 1–3 indicate a simplified nitrogen cycle in near surface environments, and ANAMMOX at in anaerobic areas - linked to reactions 33–39 in Table 1. Orange callout: highlights sulfur cycling opportunities that can occur when C&D waste is prevalent - linked to reactions 10, 27, 28 in Table 1. Green callout indicates potential for MIC and coupling to products of sulfur cycling - linked to reactions 12–14, 18–21, 23, 24, 27–32, and 40–47 in Table 1. Brown callout indicates the potential chemoautotrophic coupling of reactants across redox boundaries at the microscale when landfill ecosystems encounter exposed bedrock, as may be the case in an unlined or damaged liner - linked to reactions 10–32 in Table 1.

Landfills are constructed, nutrient rich, deep biosphere systems extending 15–100 m in depth. As such, there is no direct influence of photosynthetic activity beyond the depth of the cover soil. Thus, beyond consuming organic carbon that was produced initially by photosynthetic processes, microbial communities at depth in landfill ecosystems function without needing sunlight to drive primary production. The metabolic landscape of the deep landfill ecosystem (Figure 3) is entirely driven by chemosynthetic processes coupled to organic carbon degradation, as well as autotrophic processes that fix carbon.

Similar to other wet ecosystems, the environmental conditions that most define the deep landfill ecosystem are anaerobicity, high carbon/nutrient content, moisture, and pH (e.g., Kjeldsen et al., 2002). Metabolic processes are directed by these primary environmental controls, and further influenced by secondary or localized controls provided by spatial and temporal heterogeneity. Natural systems that could be considered analogous include, for example, wetlands (Juottonen et al., 2005; Yadav et al., 2015), thawing peat/permafrost (Wagner et al., 2005; Kip et al., 2011), estuary or lake sediments (Egger et al., 2015; He et al., 2015), marine methane seep environments (Milucka et al., 2012; Marlow et al., 2014; Sivan et al., 2014), and many terrestrial ‘deep biosphere’ systems (Lau et al., 2014; Osburn et al., 2014). Some natural wetlands offer a good comparison, with similar drying/wetting variability, rich organic deposits, and an anaerobic/aerobic interface in the soils when not fully submerged (Kolb and Horn, 2012; Wang et al., 2012).

In contrast to natural environments, the nature of managing millions of Mg of unsorted waste in engineered landfill systems introduces heterogeneity on many scales (e.g., Henneberger et al., 2015; Stamps et al., 2016). Waste inputs are layered and compacted in engineered cells with daily cover placed at the end of each day (Figure 2). Each cell can comprise <1 to >5 hectares in area with waste thickness of approximately 10 m. Individual cells may be completed in weeks to months before placement of intermediate cover. For vertical expansions, the intermediate cover may be left in place or stripped so that new waste directly overlies older methanogenic waste. Thus, in vertical aspect, as in geologic sediments, the older waste typically lies below newer unless a disruption in the ‘sedimentation’ occurs (essentially, the Law of Superposition). However, as new cells expand both vertically and horizontally over many years, the age of waste in cells in adjacent vertical sections need not be contemporaneous.

Climate variability introduces another scale of heterogeneity to landfill function, with literature suggesting that local external climate directly influences overall landfill decomposition rates from waste to methane (EMCON, 1980; Halvadakis et al., 1983; Oonk and Boom, 1995; IPCC, 1996, 2006). Climate and precipitation affect landfill anaerobicity, fluid circulation, and temperature profiles (Yesiller and Hanson, 2003; Hanson et al., 2010). Internal heat and fluid transfer processes influence biological, chemical, and geochemical processes in wastes, liners, and covers, enhancing these processes within optimum desired ranges but suppressing them in extreme temperature conditions (e.g., >65°C). Within a single landfill, the spatial variability in porosity and permeability of landfilled materials and soils (Zeng et al., 2017), and the detailed circulation of leachate may be poorly understood (see review by Muaaz-Us-Salam et al., 2019). In some cases, the limited moisture content of freshly added waste may impact the microbial communities of young landfilled waste (Staley et al., 2012).

Landfilled waste becomes anaerobic within days of burial when diverse, indigenous, hydrolytic, fermentative, acidogenic, and acetogenic microorganisms begin to degrade hydrocarbons to CO_2_, H_2_, and carboxylic acids, including acetate (Wolfe, 1979; Halvadakis et al., 1983; Barlaz et al., 1989b; Vavilin et al., 2006). This occurs at a relatively steady state rate in engineered landfills prior to methanogenesis (Stamps et al., 2016; Villar et al., 2016). In general, once methanogenesis is established, the rapid turnover of acetate, CO_2_ and H_2_ to CH_4_ can also promote the near neutral pH conditions favored by methanogens. Recent carbon isotopic studies have shown that minor amounts of methanogenesis can also occur in saturated cover soils with high organic C content (Bogner et al., 2011).

Typically, first order kinetic equations (often multicomponent) based on the degradable organic C content of landfilled waste have been applied with the kinetic constant (1/*t*) generally related to external climate. In contrast, more recent metadata analysis suggests that internal landfill conditions at current operational sites may be relatively constant at larger, whole landfill scales. As evidence, metadata comparisons using large field datasets have indicated a relatively constant rate for observed CH_4_ generation and recovery per total unit mass of buried waste. For example, Spokas et al. (2015) demonstrated a robust linear relationship for 129 full-scale California landfills between 2010 waste in place (WIP) and 2010 average CH_4_ recovery rate of approximately 125 Nm^3^ CH_4_ per million Mg waste in place (WIP). This rate was independent of external climate (MAT; MAP), as well as the age of waste and site status, suggesting more homogeneity than has been historically assumed for internal landfill processes (see also National Academies of Sciences, Engineering, and Medicine [NASEM], 2018). Indeed, IPCC national GHG inventory methods for landfill CH_4_ emissions have historically represented landfill decomposition using a 1st order model with the generated CH_4_ assumed to be partitioned to recovery, oxidation, and emissions (IPCC, 1996, 2006). In general, very few field scale validations for recovery efficiency exist: Spokas et al. (2006), studying French landfills, demonstrated recovery efficiency rates of 80–90% in covers comparable to current United States practice. One consequence of the IPCC strategy is that estimated emissions are proportional to WIP so that largest landfills (highest WIP) are always correlated with the highest emissions, which is not supported by recent California field data (Spokas et al., 2011, 2015). Moreover, well known emissions mitigation measures (thicker cover soils with higher seasonal oxidation, smaller daily working area) cannot be accounted under the current IPCC (2006) methodology (National Academies of Sciences, Engineering, and Medicine [NASEM], 2018). The major drivers for landfill methane emissions include site operational practices (thickness, composition, and timing of cover soils, installation of biogas recovery system, area of operational cell) and site specific climate, which affects transient soil gas transport and methanotrophic oxidation rates. In recent years, a process based model (CALMIM), has been developed, and field-validated for prediction of site-specific and cover specific CH_4_ emissions at any site worldwide (Bogner et al., 2011; Spokas and Bogner, 2011; Spokas et al., 2011, 2015). This model relies on diffusive bidirectional transport of CH_4_ and O_2_ through user specified cover materials as well as embedded USDA 0.5 × 0.5 climate models for 30-year average weather (GLOBAL TempSIM, RainSIM), surface energy balance (SOLARCALC), and soil temperature and moisture (STM^2^). Use of annual weather data or climate change predictions (e.g., CMIP5) is also possible. Standard outputs are cover-specific for 10 min timesteps and 2.5 depth increments over a typical annual cycle (365 days). When CALMIM was applied to 372 California landfills, it was shown that hot (>50°C), dry conditions in the Central Valley and desert regions were coupled with seasonally higher CH_4_ emissions due to reduced soil oxidation (Spokas et al., 2015).

The well documented pairing of anaerobic methanogenesis (acetoclastic or hydrogenotrophic) and aerobic methanotrophy in landfills is similar to many natural environments where degradation of organic material occurs under anaerobic conditions (Table 1, reactions 1–9; Figure 3, purple and blue highlighted processes) (Kolb and Horn, 2012; Wang et al., 2012; Marlow et al., 2014; Serrano-Silva et al., 2014). At the top of the landfill, there is a steep upward gradient from anaerobic methanogenic conditions in the buried waste to restricted aeration in cover soils, to fully aerobic conditions at the atmosphere/soil boundary. As landfill CH_4_ diffuses through cover materials it is also oxidized by methanotrophs to CO_2_ and water vapor. Area normalized methanotrophic CH_4_ oxidation rates in cover soils (i.e., g CH_4_ m^–2^ d^–1^) can vary over several orders of magnitude depending on transient soil moisture, temperature, and soil gas CH_4_ at the base of the cover (i.e., Scheutz et al., 2009; Spokas et al., 2011, 2015). Based on >2000 laboratory incubations for California cover soils of various textures, optimum conditions for methane oxidation are approximately 25–30°C with SMP (soil moisture potential) close to the water-holding capacity (Spokas et al., 2011). Importantly, as a result of long term exposure to landfill CH_4_, landfill cover soils have developed the highest reported rates for methanotrophic CH_4_ oxidation in the literature (Bogner J.E. et al., 1997; Bogner and Matthews, 1999; Bogner et al., 2011; Scheutz et al., 2009). Further, in some cases high CH_4_ oxidation rates in cover soils combined with efficient subsurface biogas recovery have resulted in documented uptake of atmospheric CH_4_ (e.g., Bogner J. et al., 1997; Bogner J.E. et al., 1997).

**TABLE 1:** Example reactions describing microbial processes in landfill ecosystems.

#	Reaction	Process	e^–^
**Carbon cycling**			
1	2CH_4_ + 3O_2_ → 2CO + 4H_2_O	Methane oxidation	12
2	CH_4_ + 2O_2_ → CO_2_ + 2H_2_O	Methane oxidation	8
3	CH_4_ + NO_3_^–^ + 2H^+^ → NH_4_^+^ + CO_2_ + H_2_O	Methane oxidation	8
4	CH_4_ + NO_2_^–^ + 2H^+^ → NH_4_^+^ + CO + H_2_O	Methane oxidation	6
5	3H_2_ + CO → CH_4_ + H_2_O	Methanogenesis	6
6	4H_2_ + CO_2_ → CH_4_ + 2H_2_O	Methanogenesis	8
7	2 NH_4_^+^ + CO → N_2_ + CH_4_ + 2 H^+^ + H_2_O	Methanogenesis	6
8	3 N_2_ + 5 CO + 13 H_2_O → 6 NO_3_^–^ + 5 CH_4_ + 6 H^+^	Methanogenesis	30
9	CH_3_COOH_(acetic acid)_ → 2CO_2_ + 4H_2_	Acetate breakdown	
**Iron/sulfur/carbon coupled cycling**			
10	H_2_S + 2 O_2_ → SO_4_^2–^ + 2 H^+^	Sulfide oxidation	8
11	4 H_2_S + O_2_ + 2 Fe^2+^ → 2 FeS_2 Pyrite_ + 4 H^+^ + 2 H_2_O	Sulfide oxidation	4
12	12 Fe^2+^ + CO_2_ + 14 H_2_O → 4 Fe_3_O_4 Magnetite_ + CH_4_ + 24 H^+^	Ferrous iron oxidation	8
13	8 Fe^2+^ + CO_2_ + 10 H_2_O → 4 Fe_2_O_3 Hematite_ + CH_4_ + 16 H^+^	Ferrous iron oxidation	8
14	2 H_2_S + Fe^2+^ → H_2_ + FeS_2 Pyrite_ + 2 H^+^	sulfide oxidation	2
15	H_2_S + Fe_3_O_4 Magnetite_ + 6 H^+^ → S + 3 Fe^2+^ + 4 H_2_O	Sulfide oxidation	2
16	2 H_2_S + Fe_2_O_3 Hematite_ + 2 H^+^ → FeS_2 Pyrite_ + Fe^2+^ + 3 H_2_O	Sulfide oxidation	2
17	2 H_2_S + 2 FeOOH_Ferrihydrite_ + 2 H^+^ → FeS_2 Pyrite_ + Fe^2+^ + 4 H_2_O	Sulfide oxidation	2
18	FeS_2 Pyrite_ + 8 H_2_O → 2 SO_4_^2–^ + Fe^2+^ + 7 H_2_ + 2 H^+^	Pyrite oxidation	14
19	4 FeS_2 Pyrite_ + CO_2_ + 8 H^+^ → 8 S + 4 Fe^2+^ + CH_4_ + 2 H_2_O	Pyrite oxidation	8
20	4 FeS_2 Pyrite_ + 7 CO_2_ + 18 H_2_O → 8 SO_4_^2–^ + 4 Fe^2+^ + 7 CH_4_ + 8 H^+^	Pyrite oxidation	56
21	FeS_2 Pyrite_ + 2 H^+^ → 2 S + Fe^2+^ + H_2_	Pyrite oxidation	2
22	CH_4_ + Fe^3+^ + 2H_2_O → CO_2_ + 2Fe^2+^ + 3H_2_ + 2H^+^	Ferric iron reduction	6
23	H_2_ + FeS_2 Pyrite_ + 2H^+^ → Fe^2+^ + 2H_2_S	Pyrite reduction	2
24	H_2_ + 2 FeS_2 Pyrite_ + 4 H_2_O → 2 FeOOH_Ferrihydrite_ + 4 H_2_S	Pyrite reduction	4
25	CH_4_ + 4FeS_2 Pyrite_ + 8H^+^ + 2H_2_O → 8H_2_S + 4Fe^2+^ + CO_2_	Pyrite reduction	8
26	CH_4_ + 8 FeS_2 Pyrite_ + 14 H_2_O → 4 Fe_2_O_3 Hematite_ + CO_2_ + 16 H_2_S	Pyrite reduction	16
27	4H_2_ + SO_4_^2–^ + 2H^+^ → H_2_S + 4H_2_O	Sulfate reduction	8
28	7 H_2_ + 2 SO_4_^2–^ + Fe^2+^ + 2 H^+^ → FeS_2 Pyrite_ + 8 H_2_O	Sulfate reduction	14
29	4CH_4_ + 3SO_4_^2–^ + 6H^+^ → 3H_2_S + 4CO + 8H_2_O	Sulfate reduction	24
30	CH_4_ + SO_4_^2–^ + 2H^+^ → H_2_S + CO_2_ + 2H_2_O	Sulfate reduction	8
31	7 CH_4_ + 8 SO_4_^2–^ + 4 Fe^2+^ + 8 H^+^ → 7 CO_2_ + 4 FeS_2 Pyrite_ + 18 H_2_O	Sulfate reduction	56
32	8 Fe^2+^ + SO_4_^2–^ + 12 H_2_O → 8 FeOOH_Ferrihydrite_ + H_2_S + 14 H^+^	Sulfate reduction	8
**Nitrogen cycling**			
33	2 NH_4_^+^ + 3 O_2_ → 2 NO_2_^–^ + 4 H^+^ + 2 H_2_O	Nitrification I	12
34	2 NO_2_^–^ + O_2_ → 2 NO_3_^–^	Nitrification II	4
35	H_2_ + NO_3_^–^ → NO_2_^–^ + H_2_O	Denitrification I	2
36	3H_2_ + NO_2_^–^ + 2H^+^ → NH_4_^+^ + 2H_2_O	Denitrification II	6
37	3H_2_ + N_2_ + 2H^+^ → 2NH_4_^+^	Nitrogen fixation	6
38	H_2_S + NO_3_^–^ + H_2_O → SO_4_^2–^ + NH_4_	Nitrate reduction	8
39	NH_4_^+^ + NO_2_^–^ → N_2_ + 2 H_2_O	Nitrite reduction	3
**Microbially induced corrosion (MIC) – includes reactions 12–14, 18–21, 23, 24, 27–32**			
40	4 Fe^2+^ + O_2_ + 6 H_2_O → 4 FeOOH_Ferrihydrite_ + 8 H^+^	Ferrous iron oxidation	4
41	2 Mn^2+^ + O_2_ + 2 H_2_O → 2 MnO_2 Pyrolusite_ + 4 H^+^	Manganese oxidation	4
42	Mn^2+^ + FeS_2 Pyrite_ + 2 H_2_O → MnO_2 Pyrolusite_ + Fe^2+^ + 2 H_2_S	Manganese oxidation	2
43	Mn^2+^ + 2 FeS_2 Pyrite_ + 5 H_2_O → MnO_2 Pyrolusite_ + Fe_2_O_3 Hematite_ + 4 H_2_S + 2 H^+^	Manganese oxidation	4
44	4 CO + SO_4_^2–^ + 2 H^+^ → H_2_S + 4 CO_2_	Sulfate reduction	8
45	7 CO + 2 SO_4_^2–^ + Fe^2+^ + 2 H^+^ → 7 CO_2_ + FeS_2 Pyrite_ + H_2_O	Sulfate reduction	14
46	7 H_2_S + SO_4_^2–^ + 4 Fe^2+^ → 4 FeS_2 Pyrite_ + 6 H^+^ + 4 H_2_O	Sulfate reduction	7
47	CH_4_ + 8FeOOH _Ferrihydrite_ + 16H^+^ → 8Fe^2+^ + CO_2_ + 14H_2_O	Ferrihydrite reduction	8

*The number of electrons transferred in each reaction is listed at right. Reactions are listed with aerobic processes first, followed by anaerobic processes.*

Methane can further be removed by anaerobic microbial oxidation (AOM), sometimes coupled with nitrate, nitrite, sulfate, ferric iron, or minerals (e.g., Milucka et al., 2012; Sivan et al., 2014; Egger et al., 2015) (Table 1, reactions 3, 4, 22, 25, 26, 29, 30, 31). Huber-Humer (2004) demonstrated *in situ* anaerobic CH_4_ oxidation in anoxic laboratory soil columns amended with high sulfate biosolids. Other potential pathways for anaerobic methane oxidation have been demonstrated in a variety of natural settings (Wang et al., 2012, 2019; Yan et al., 2018; Shen et al., 2019; Vigneron et al., 2019), but are as yet undocumented in landfills.

As in other wet soil environments, sulfate reduction may be coupled to organic carbon degradation, methane, and hydrogen consumption (Table 1, reactions 27–32), and thus both support (via CO_2_ production) and compete with methanogenesis (e.g., Gurijala and Suflita, 1993; Visser et al., 1993; Long et al., 2016). Addition of sulfate-rich substrates to the landfill ecosystem, such as gypsum wallboard in construction and demolition (C&D) waste, use of high sulfate, local cover soils or landfill development in S rich geologic settings (e.g., old mine pits with sulfide mineralization weathered to sulfates) may locally boost the activity of sulfate reducing bacteria (SRB; Figure 3, orange highlighted processes) (Fairweather and Barlaz, 1998; Sun and Barlaz, 2015). Laboratory studies of simulated landfill settings suggest that sulfate reduction can suppress methanogenesis where leachate sulfate levels are >500 mg L^–1^ (Moreau-Le Golvan et al., 2003), comparable to anaerobic natural environments, such as shallow marine settings where methanogenesis may be suppressed until sulfate is depleted. While sulfate reduction and methanogenesis can occur concurrently in landfill ecosystems, methanogenesis was found to be the dominant electron sink in landfill reactors in the presence of sufficient organic matter, even when there was an excess of sulfate from construction and demolition debris (Fairweather and Barlaz, 1998). Abundant sulfate reduction may result in a drop in localized fluid and leachate pH as sulfuric acid is produced. If an available oxidant is present to oxidize the H_2_S, acid build up may be prevented. Surface ecosystems conveniently provide oxygen to return H_2_S to sulfate in a simple sulfur cycle loop (Table 1, reactions 10, 11). However, in anaerobic environments such as landfills, H_2_S can be oxidized by mineral surfaces, ferric iron, or nitrate/nitrite (Figure 3, green and brown highlighted processes; Table 1, reactions 14, 15, 16, 17, 38). In some sites, empirical evidence has suggested that sulfate reducing consortia in basal collection systems have driven secondary carbonate deposition (e.g., Maliva et al., 2000).

The iron, sulfur, and carbon cycles are closely associated in landfill environments (Kwon et al., 2016). Availability of iron as a substrate is likely to be highly heterogeneous in landfill environments, variable with waste chemistry, the landfill geologic setting, the composition of local cover soils and permitted alternative covers, as well as variable internal pH and redox conditions. Microbially induced corrosion (MIC) of iron can transform iron substrates even in higher pH systems (Figure 3, green highlighted processes; Table 1, reactions 12–14, 18–21, 23, 24, 27–32, and 40–47). Many organisms have been implicated in MIC (see reviews by Zarasvand and Rai, 2013 and Kip and van Veen, 2015), but well recognized groups are SRB (e.g., Enning and Garrelfs, 2014, manganese/iron/sulfur oxidizing bacteria (Rajasekar et al., 2005), iron reducing bacteria (Herrera and Videla, 2009), and acid producing bacteria (Li et al., 2008), as well as methanogenic archaea when in contact with the metal in question (Enning and Garrelfs, 2014).

Nitrogen cycling processes are also active in landfill ecosystems (Figure 3, white highlighted processes; Table 1, reactions 33–37). Landfill leachate typically contains high concentrations of NH_4_^+^ (Kjeldsen et al., 2002). Interestingly, empirical data from landfill biogas recovery systems have also periodically documented N_2_ recovered from deep landfills without any possibility of atmospheric input, suggesting that ANAMMOX processes may be naturally occurring in deep landfill settings (Figure 3, white highlighted processes; Table 1, reaction 39). Field data have demonstrated that, in landfill cover soils, soil gas N_2_O can be elevated in the semi-aerobic portion of final covers with available NO_3_^–^; however, N_2_O emissions are typically within the wide range measured for natural and agricultural soils (Bogner and Matthews, 1999; Bogner et al., 2011). Börjesson and Svensson (1997) showed elevated rates of N_2_O emissions in cover soils amended with high N biosolids.

As the needs, resources, norms, and practices of society have changed over time, so has the composition of landfilled waste. Microbial processes in modern landfills must be put in the context of changing societal practices at local to international levels (e.g., Rathje and Murphy, 1992). Components such as hazardous wastes, plastics, garden waste, construction and demolition wastes, industrial wastes, recyclable materials, and the presence of various contaminants of emerging concern (CECs) have varied substantially through time, including both potential substrates for microbial metabolism as well as potential inhibitors of microbial growth (Stamps et al., 2016). For example, CECs may include pharmaceuticals or other wastes that confine or inhibit microbial community function (Andrews et al., 2012; Masoner et al., 2014, 2016; Wang et al., 2015; Wu et al., 2015, 2017; Song et al., 2016). During the last two decades, there has been increased landfill disposal of personal care products (solid deodorants, hair conditioners, etc.) with elevated siloxanes and silanes which are volatile at internal landfill conditions and occur as trace constituents of landfill gas, resulting in solid siliceous deposits which greatly reduce the operational life of biogas engines (Wheless and Pierce, 2004; Nair et al., 2013). A current concern is landfilled ash from aluminum production (‘dross’) and waste incineration where dangerously high internal temperature conditions (>100°C) can result from internal abiotic exothermic reactions in the waste mass (Benson, 2017; Hao et al., 2017b). Empirical biogas data (% v/v) from such sites (e.g., Jafari et al., 2017a) indicate that high temperatures suppress methanogenesis, as evidenced by decreased CH_4_ coupled with increased CO_2_, H_2_, and measurable CO (presumably from microbial pathways: Hoshino and Inagaki, 2017; Robb and Techtmann, 2018).

These few among many examples serve to highlight the vastness of the research remaining in landfill microbiology, and how far this research can extend beyond understanding the methanogenic and methanotrophic populations. In short, despite being engineered ecosystems, the taxonomic, genetic, and functional diversity of the microbial communities living in the highly heterogeneous landfill environments are just as complex and poorly understood as microbial ecosystems occurring in natural environments.

## An Historic Look at Landfill Microbiological Research

To set the context of what is known about landfill microbiology and ecology, and the potential impact of modern and high-throughput sequencing technologies on this knowledge base, we will first review the main trends identified during the 1980s–1990s. Early research on landfill microbiology focused on understanding the conditions that controlled methanogenesis and the organisms that were found to be responsible for organic degradation in landfills. This work was primarily accomplished in laboratory settings, relying on culture dependent methodologies. Culturing work found that all trophic groups required for refuse methanogenesis are present in fresh refuse (cellulolytics, acetogens, and methanogens), including both acetoclastic and hydrogenotrophic methanogens (Barlaz et al., 1989b; Mormile et al., 1996). Barlaz et al. (1989b) described the process of refuse decomposition from their laboratory experiments as occurring in four phases: (1) the aerobic phase in which oxygen and nitrate are depleted; (2) the anaerobic acid phase characterized by accumulation of carboxylic acids and a decrease in pH; (3) the accelerated methane production phase in which methanogen population and methanogenesis increase and carboxylic acids decrease; and (4) the decelerated methane production phase.

Early work determined that increased moisture content was found to be more a favorable environment for waste breakdown and methanogenesis (Bogner, 1990; Gurijala and Suflita, 1993). Moisture content can benefit the solubilization and distribution of substrates and nutrients and the dilution of toxic substances (Barlaz et al., 1987). However, it is possible that additional moisture may also solubilize substances that inhibit methanogenesis, such as alternative electron acceptors (Gurijala and Suflita, 1993). Moisture alone does not account for all variation in methane production rates, and there is evidence that even a refuse-moisture content of >50% will not ensure methane production (Barlaz et al., 1987; Gurijala and Suflita, 1993). Several studies explored the effect that additional moisture may have on landfill pH, and found that additional moisture may stimulate the initial fermentation processes leading to hydrolysis of cellulose and polymers and lead to an accumulation of carboxylic acids, depressing the pH of the waste and inhibiting methanogenesis (Pohland, 1980; Barlaz et al., 1987; Gurijala and Suflita, 1993). However, the depression of pH due to accumulation of organic acids and subsequent methanogenesis inhibition was also observed regardless of additional water content (Barlaz et al., 1987; Mormile et al., 1996). It was hypothesized that active and well established methanogenic populations could tolerate and function under acidic pH conditions in landfill ecosystems (Kasali et al., 1988). This was later supported by work from Ladapo and Barlaz (1997), who were able to isolate acid tolerant methanogens that could grow at a pH of 5.5.

There was also an early focus on hydrogen as a critical intermediate in landfill ecosystems. Hydrogen is produced by both the hydrolytic and acetogenic bacteria and it is a substrate for methanogens (Barlaz et al., 1987). The accumulation of hydrogen was found to be an indication of an imbalance in the microbial population (Barlaz et al., 1987). This was supported by Mormile et al. (1996) who suggest utilizing H_2_ concentrations to monitor landfill status in high temperature landfills. They found that samples with higher rates of methanogenesis had lower hydrogen concentrations and more neutral pH values than those with lower rates of methanogenesis because H_2_ was rapidly consumed by methanogens or other organisms, and an apparent steady state was achieved. Other important intermediates are organic acids. It has been found that high methane producing samples have low organic acid concentrations, while low methane producing samples accumulated high concentrations of organic acids during the initial fermentation reactions (Boone and Mah, 1987; Barlaz et al., 1989a; Mormile et al., 1996).

## The Molecular Microbiology ‘Revolution’ – What Sanger Sequencing Revealed About Landfill Ecology

In the late 1990s and into the early 21st century, the use of Sanger sequencing technology vastly expanded what was known about landfill microbiology. The ability to move beyond culture dependent work, by applying 16S rRNA targeted PCR followed by molecular cloning and Sanger sequencing (or a variety of fingerprinting methods) directly to samples, allowed a first peek at the potential complexity and proportions of archaea and bacteria in landfill solids and leachates and informed experimental work. While this review will move beyond these now largely outdated methods, it is vital to understand the strides that were made in landfill microbial ecology during this time period, in order to identify knowledge gaps.

### Types of Methanogens Present, and Impact of Environmental Conditions

Early attempts to identify the diversity of methanogens in landfill solids using molecular cloning and Sanger sequencing techniques focused largely on landfills in Asia. These studies sought to understand the influence of depth/age of landfilled materials on methanogenic diversity (e.g., Chen et al., 2003a, b), and the relationship between diversity and type of dominant landfilled materials (e.g., Uz et al., 2003; Tang et al., 2016). These works found evidence for a community succession of methanogens and bacteria that changed as the primary source of carbon matured. Statistical treatment of these limited data indicated that depth, landfill age, total carbon, total phosphorus, pH, and moisture explained community variation, with pH and total phosphorus being the most important controlling factors. These data showed that a wide range of environmental conditions influenced, or co-influenced microbial diversity of landfill ecosystems. The first analyses of beta diversity within individual landfills and across landfill locations were presented with Sanger sequencing results. Laloui-Carpentier et al. (2006) noted that across various studies of both leachate (Huang et al., 2003; Laloui-Carpentier et al., 2006) and solid waste from MSW landfills, regardless of the nature of the starting materials, sampling method, DNA extraction technique, or PCR protocol, the major groups of methanogens found in MSW landfills worldwide belong to the genera *Methanosarcina*, *Methanosaeta*, *Methanoculleus*, and *Methanofollis*. It has also been observed that the bacterial and archaea communities of leachate and solids are statistically different, suggesting that different members of the community are supported by leachate than by solid wastes (Staley et al., 2012).

Despite these advances in sequencing technology, contemporaneous experimental work hinted that critical details about landfill microbial ecosystems were being missed with 16S rRNA approaches. Culture dependent studies revealed that methanogens cultured in the laboratory could thrive at pH values < 7 and that populations of methanogens shifted as acid production changed (e.g., Ladapo and Barlaz, 1997; Staley et al., 2011). Qu et al. (2009) provided means for cross referencing sequencing data with stable isotopes of carbon to challenge what was known about hydrogenotrophic and acetogenic methanogenesis in landfills, suggesting that Methanosarcinaceae may adjust metabolic function *in situ* as landfill conditions shift. In short, while Sanger sequencing based methodologies revealed much about community composition in landfills, they also lack the ability to robustly investigate microbial functions under various environmental conditions. Partnered with experimental efforts, it became clear that a deeper examination of the dynamics of methanogenesis in landfill ecosystems was needed.

### Methanotrophs in Cover Soils

Methane oxidizing bacteria (MOB) were also early targets of Sanger sequencing efforts, as their influence on greenhouse gas emissions was recognized. Studies of the diversity of MOB in landfills reported conflicting findings on the dominant members of the community, with some reporting abundance of both Type I and Type II MOB (Cébron et al., 2007; Gebert et al., 2009; Lin et al., 2009; Han et al., 2016) and others reporting a dominance of one or the other group (Chen et al., 2007; Héry et al., 2008; Henneberger et al., 2012). In general, trends seemed to indicate that Type I MOB dominated in high nutrient, high oxygen environments (Liebner et al., 2009; Dumont et al., 2011; Graef et al., 2011; Krause et al., 2012), and Type II MOB preferred reduced oxygen and nutrient conditions, often had a higher presence in communities at greater depth in the cover soil, and might effectively be dormant until nutrient limited conditions formed (Shrestha et al., 2010; Bodelier et al., 2012; Krause et al., 2012) or after a system disturbance (Ho et al., 2011; Ho and Frenzel, 2012). In reality, it is likely a consortium of both Type I and Type II methanotrophs provide functional redundancy in the system, allowing adaptation to fluctuating environmental conditions (e.g., Tajima et al., 1999; Mukred et al., 2008).

While sequencing of 16S rRNA gene diversity in landfill cover soils may give an overall picture of community diversity and structure, the concurrent sequencing of transcribed DNA (cDNA) raised concerns that 16S rRNA gene diversity analysis misses key details concerning which organisms are the most active in the community. The diversity and activity (by reverse transcription PCR) of the 16S rRNA gene and the functional genes *mmoX*, *pmoA*, and *mxaF* were examined in a landfill cover soil to 40 cm depth (Chen et al., 2007). Both Type I and II methanotrophs were found to coexist in these soils, as in other reports (Börjesson et al., 2004; Stralis-Pavese et al., 2006; Cébron et al., 2007; Gebert et al., 2009; Lin et al., 2009; Han et al., 2016). *Methylobacter* strains were the major Type I organisms, and *Methylocystis* and *Methylocella* spp. were the major Type II organisms in the 16S rRNA gene clone library. However, analysis of transcribed DNA revealed that *Methylomonas*, a minor member of the Type I population, was more active in the population at the time of sampling, and *Methylocystis* (a major percentage of the Type II population) was not active in proportion to the numbers found in the 16S rRNA clone library. Further, *pMMO* appeared to be responsible for methane oxidation in this soil, suggesting Type II *Methylocella* sp. were not actively consuming methane at the time of sampling. The addition of examining genes that are active at the time of sampling may provide a deeper understanding of community dynamics in landfill cover soils.

### Bacteria vs. Archaea

With much of the focus centering on methanogenic and methanotrophic populations, and efficiency of methane production, bacterial populations in MSW leachate were not initially studied as widely. Work by Huang et al. (2002, 2003, 2004, 2005) showed that populations of both archaea and bacteria were present in leachate, and that both should be studied in conjunction in order to have any insight into MSW microbial community dynamics. Huang et al. (2003, 2004, 2005) provided an early analysis of both bacterial and archaea diversity on two MSW landfills in China, finding that the bacterial community was unexpectedly diverse, with >103 distinct sequence types and the majority of retrieved 16S rRNA bacterial sequences belonging to uncultivated species. This first look at bacteria in a landfill leachate gave early indication that much remained to be known about the microbial dynamics in landfill ecosystems.

Due to the limited number of examples of work that explored both bacterial and archaea communities of solids in MSW landfills from this technological time frame (e.g., Krishnamurthi and Chakrabarti, 2013), it is not reasonable to compare and contrast the microbial communities of solids with those in the leachates. However, experimental work probed the problem of whether leachate samples can reasonably represent the microbial community of an MSW landfill as a whole. Staley et al. (2012) found that attention to the age of the waste is paramount to drawing conclusions concerning microbial activity. The subject of community succession was again tested experimentally by Staley et al. (2018), targeting different stages of waste decomposition, where it was found that community succession of both bacteria and archaea was driven by pH (rather than acetate concentrations). Direct validation of these findings in landfill environments will be essential in the future.

## Changing Previous Assumptions Using Modern Techniques – A ‘Second Revolution’ in Molecular Microbiology

A ‘second revolution’ in molecular biology is currently occurring, as sequencing technology improves, producing larger datasets that require application of advanced bioinformatics and statistical approaches. Widespread access to high throughout sequencing methods now allows analysis of DNA and RNA so cheaply that experimental design has changed in response. Methods used to screen samples before sequencing to maximize results, such as Denaturing Gel Gradient Electrophoresis (DGGE), Restriction Fragment Length Polymorphism (RFLP) and other similar ‘fingerprinting’ methods, are largely no longer needed. Many laboratories are now able to sequence all their samples with replicates, for less than the cost of sequencing only the most relevant samples a decade ago. Next generation sequencing (NGS) platforms have been evolving for the last decade, from early short read length platforms (e.g., 454 ‘tag’ pyrosequencing or Ion Torrent technologies) to current short read Ilumina platforms (Hiseq, Miseq, Miniseq). Emerging long read length technologies such as Nanopore sequencing (e.g., Oxford MinION) or by Pacific Biosciences hold promise for even more information dense sequencing (see reviews of NGS sequencing, Goodwin et al., 2016; Patel et al., 2018; Mantere et al., 2019). NGS technologies produce so much data per sample that the problem has moved from the high expense of sequencing to the expense of hiring personnel to process hundreds of gigabytes of data. High throughput sequencing platforms have facilitated re-evaluation of the microbial ecology of a wide array of natural and artificial ecosystems. Examination of the 16S rRNA gene diversity is still the standard approach for community analysis, and amplicon sequencing of community DNA has had a major impact on views of the diversity of taxa and community structure in many ‘dark’ natural systems such as subglacial, permafrost soils, and examples from the subsurface biosphere (e.g., Hamilton et al., 2013; Jansson and Taş, 2014; Hug et al., 2016; Crespo-Medina et al., 2017; Momper et al., 2017).

In addition to amplicon sequencing of 16S rRNA genes, the current affordability of high throughput sequencing has also increased the use of ‘omics’ methodologies. ‘Omics,’ or ‘meta’ analysis refers to the analysis of full community genomic, transcriptomic, proteomic, or lipidomic analysis. Metagenomic analysis^3^ provides partial or full genomic information of taxa in a community, as determined through sequencing of short or long reads of environmental/community genomic DNA. Similarly, following reverse transcription, metatranscriptomics provides a look at what RNA transcripts are present in a given sample at the time of sampling and preservation. Combined, high throughput sequencing and -omics related analytical methodologies give microbial ecology researchers the ability to sample more exhaustively, with replicate analyses, in addition to probing the functional diversity of a microbial community at greater depths than have been possible using Sanger sequencing or culturing based approaches (Hess et al., 2011; Miao et al., 2015a, b; Nobu et al., 2015). Occasionally the use of an -omics methodology has revealed key ecological functions of previously uncultivated microorganisms, allowing enough insight into community function to enable the enrichment and isolation of new cultivars (e.g., Tyson et al., 2005; José León et al., 2014). The ability to link community functional diversity to environmental conditions is rapidly improving, and truly represents a new revolution in microbial ecology (De Vrieze et al., 2018).

Knowledge of the identity, abundance, and function of MSW landfill microbiota is still limited, as earlier studies were restricted by necessity to a limited number of isolates and clones that did not represent the *in situ* diversity and dynamics of the microbial population in landfills. Despite the availability of high throughput sequencing methods, as of this writing they are still applied only sparingly in the study of landfill soils and leachate. An extensive literature search revealed fewer than ten examples of studies that applied next generation sequencing techniques directly to field obtained samples of landfill soils, solids, or leachate. A few additional works utilized them in laboratory settings, in enrichments or batch culturing. Below, we highlight literature that assesses microbial diversity and function using NGS based technologies, with particular attention to areas in which these technologies have improved our previous understanding.

### Methanotrophs in Cover Soils: Invigorated Interest Due to GHG Concerns

A surge of new interest in landfill cover soils and microbial controls on methane emissions has encouraged an application of high throughput sequencing methods. As discussed above, the proportions of Type I vs. Type II methanotrophs in cover soils, and the environmental factors that control them, have been subjects of contention (e.g., Ho et al., 2016). Yargicoglu and Reddy (2017) investigated the abundance and distribution of methylotrophic bacteria in cover soils with and without biochar amendment. Methylotrophic (Alphaproteobacteria) and methanotrophic bacteria, primarily belonging to the Type I group (Gammaproteobacteria, *Methylomonas, Cenothrix*), dominated over Type II methanotrophs and were shown to be more abundant in sample sites with more abundant methane. No statistically relevant correlation was found to other environmental and spatial parameters. The abundance of methanotrophs varied from 1.5 to 68% of all identified taxa across all sites sampled. Type I methanotrophs have been previously reported to be more abundant in areas of high methane. However, as demonstrated in Yargicoglu and Reddy (2017), high throughput sequencing allows statistical approaches to lend weight to conclusions. Further, these results show that, at least at this specific location, non-methanotrophic microorganisms compose 32–96% of cover soil community organisms, highlighting the extent to which contributions of other biogeochemical cycles are unknown.

### Revealing the Complexity of Methanogen Diversity and Succession

The 454 tag pyrosequencing method was applied to a large study directly examining the archaeal communities of landfill leachates that were removed via a drilling/pumping method directly from eleven locations within six landfills in China (Song et al., 2015). With tens of thousands of sequencing ‘reads’ per sample, and hundreds of operational taxonomic units (OTUs) identified, the estimated diversity of the samples was several orders of magnitude higher than had been previously determined using molecular cloning plus Sanger sequencing approaches. With ‘deeper’ sequencing data, the authors were able to determine that the archaeal community was unique at each location, as were the chemical compositions of the leachate sampled. While some dominant taxa, such as *Methanoculleus*, are familiar from older literature, other taxa found have seldom been reported as major taxa. These include the *Methanothermobacter* and *Methanocorpusculum*, and the authors hypothesize that the direct sampling technique, in addition to the more thorough diversity survey, were responsible for detecting these taxa as major players in the landfill community. While the authors further speculate that they are syntrophic with acetate oxidizing bacteria, their specific roles are thus far unknown (Song et al., 2015). This would further explain the finding that hydrogenotrophic methanogens were responsible for the majority of the methane production in these locations (Song et al., 2015), supporting previous reports (Huang et al., 2002, 2003; Chen et al., 2003a, b; He et al., 2007) that Asian landfills with high putrescible content are rich in hydrogenotrophic methanogens. Lastly, the order of succession of methanogens as the age of the refuse increased and environmental conditions shifted was not consistent with previous reports (Bogner et al., 1996; Barlaz, 1998; Kjeldsen et al., 2002), perhaps indicting that the dynamics of methanogenic succession is not as well understood as previously thought.

### Expanding Our View of Diversity and Influencing Factors

While much of our previous discussion has focused on landfills in Asia and Europe, the first comprehensive survey of MSW landfills in the United States was performed on leachates by Stamps et al. (2016), and included the use of high throughput sequencing. These landfills varied in host climate zone, management practices, total waste composition, and other geophysical and geochemical parameters and came from sixteen different states across the country. This work utilized the Illumina Miseq platform (arguably the most frequently used in similar applications at the time of writing), retrieving over a million high quality sequence reads from the nineteen landfills studied, to which statistical analyses were then applied. The authors showed that the nineteen landfills clustered by microbial composition into four distinct groups (with a couple of outliers), each of which had a different dominant taxa. Effectively, each leachate microbiome was unique. Further, the geographic region hosting the landfills had the most influence on this diversity, where the availability of sulfate, the evapotranspiration rate, and the age of the waste were the factors that best explained the distribution of microbial compositions. Importantly, Stamps et al. (2016) also identified a potential novel group of methanogens, the *Methanomethylophilu*s, that were dominant among the methanogens found here but seldom reported in previous literature. This deep sequencing of US landfills followed by statistical analysis enabled Stamps et al. (2016) to make a direct comparison with other microbiomes for which high throughput sequencing is available, and they showed that MSW landfills are a completely unique microbiome unlike any in other natural or engineered ecosystems.

The identification of a new group of potential methanogens (Stamps et al., 2016), in addition to reports of orders of magnitude more diversity than shown previously (Song et al., 2015) and a landfill metagenome composed of up to 30% unidentified taxa (Gupta et al., 2017), show that the true diversity and function of microorganisms in landfill microbiomes is not yet known. Some have postulated that the new diversity estimates are made up primarily of rare taxa, the function of which is unknown or suspected to not be essential to the landfill microbiome. However, rare taxa have also been identified as providing duplicate/redundant function that will help the ecosystem survive environmental hardship by supplying functional redundancy (Köchling et al., 2015).

While microbial communities in experimental approaches are often less diverse than the source system, high throughput sequencing is already having an impact on the efficacy of experiments as well as the types of questions that can be reasonably answered in laboratory settings. Topics such as best practices for leachate circulation, optimization of methane production, and which organisms are responsible for degrading specific compounds are being addressed with high throughput sequencing technologies (Bareither et al., 2013; Fei et al., 2015; Gupta et al., 2017; Ransom-Jones et al., 2017). Effectively, when the cost of obtaining sequence information is no longer a limiting factor in experimental design, new avenues for exploration are made available. While the full report is not yet available, the announcement of a metagenome from leachate of a landfill in New Delhi, India will allow examination of specific functional genes present in the leachate (the ‘functional capacity’ of the leachate microbiome) in addition to giving a vastly superior picture of the diversity and proportions of microorganisms in that microbiome (Gupta et al., 2017). Ransom-Jones et al. (2017) examined cellulolytic bacteria in both landfill leachate and via experimental enrichments, using 16S rRNA amplicon surveys as well as metagenomic analyses. Such paired techniques provide the ability to dig into the details of specific landfill functions, under specified conditions. Ransom-Jones et al. (2017) found that both Metagenome and 16S rRNA gene amplicon sequencing demonstrated the dominance of *Firmicutes*, *Bacteroidetes*, *Spirochaetes*, and *Fibrobacteres* in the landfill cellulolytic community. In previous studies, the diversity of the cellulose degrading community has been underrepresented in general, and specifically the Clostridiales have possibly been mistakenly identified as the primary cellulose degraders in landfill ecosystems, partially due to methodological biases. Among these cellulose degrading bacteria, functional metagenome analysis found 8,371 carbohydrate active enzymes (CAZy) (Ransom-Jones et al., 2017). Unlike previous studies using older, or less complete sequencing methods, the Fibrobacteres were not only found in this landfill system, but they were found to have a cellulase system - extending the ecological range of *Fibrobacter* cellulose systems to landfills. This report also represents the first detection of the major components of a cellulase system in Bacteroidetes (Ransom-Jones et al., 2017). Together, these findings suggest that multiple mechanisms of biomass degradation are present in landfill microbiomes, and specifically that cellulolytic members have been poorly represented in 16S rRNA databases to date.

## Implications of Advanced Sequencing for Landfill and Solid Waste Management

The implications of the above discussion are that not only has our understanding of the complexity and extent of microbial diversity in landfill ecosystems been lacking, but that NGS sequencing has the potential to mitigate discrete landfill problems, especially if paired with experimental or field based research. Not all landfills perform as expected. More complete understanding of the dynamics of landfill microbiology could also assist management practices as we move forward (Staley et al., 2018), especially with regard to increasing source separation (i.e., diversion of food waste to anaerobic digestion) or pre-treatment of waste before it reaches a landfill via mechanical and biological treatment (MBT) which is widely practiced in Europe. While most modern landfills do not typically experience leaking leachate issues, contamination from aging landfills is still a concern. Availability of clean drinking water will become increasingly scarce in the growing geopolitical reality, and contamination of fresh water sources is a major concern worldwide. Given the immediate necessity to track and regulate the production and consumption of CH_4_ in landfill settings, the impact of future climate change on methanotrophy in landfill cover soils is of paramount concern. Finally, with regard to the availability of future fuel resources, the ability to harness additional biogas and biofuels from MSW will be of increasing interest. Current and developing sequencing technologies have already influenced existing knowledge of these areas of concern.

### Deeper Understanding of CH_4_, N_2_O and H_2_S Production and Emissions in Landfills, Anaerobic Digestion, and Composting Environments

Landfill management has long been focused on the control of gases and leachate, both to optimize degradation of waste and to reduce emissions of aqueous contaminants, methane and hydrocarbon trace gases which contribute to urban ozone formation. However, to date, there is little direct knowledge regarding the spatial and temporal diversity of microbial populations in landfill settings. Although recent work has examined anaerobic microbial consortia in landfill settings and aerobic microorganisms in cover materials, there has been little direct linkage to organic C degradation processes and products. For example, Slezak et al. (2015) showed that even small amounts of aeration and recirculation of leachate can produce significantly more CO_2_ (∼19%) than non-circulated and anaerobic controls.

Diversion of organic waste from United States landfills continues to increase^4^. More than 25 US states currently require source separation of garden waste, typically to aerobic composting operations sited concurrently with landfill sites. In California and other states, high levels of source separation and treatment of food waste via composting and, increasingly anaerobic digestion, are being phased in under various state and urban initiatives. For example, under California SB 1383 (2016) addressing reductions in emissions of CH_4_ and other ‘short lived climate pollutants’ below 2014 base levels, California is targeting a 50% reduction in landfilling of organic waste by 2020 and a 75% reduction by 2025^5^.

Applying advanced sequencing methods and transcriptome based methods to experimentation could reveal which microbial processes were encouraged under specific process conditions. For example, application of high throughput sequencing of 16S rRNA to microbial communities subjected to different pretreatment conditions (oxygen concentrations between 0 and 21%) of food waste revealed that important shifts in the microbial community occurs both in the initial phases of pretreatment and after conditions were established (Fisgativa et al., 2018). Further, co-registration of community composition with environmental analyses showed that while the pretreatment was being established during day one, oxygen was not the only environmental parameter to impact community composition (Fisgativa et al., 2018). Fisgativa et al. (2018) established that higher degrees of aeration both rapidly and deeply impacted the microbial community, shifting the organisms from an initial dominance of lactic acid bacteria toward VFA consumers, as the conditions favored a decrease in simple carbohydrates and VFA, and an increase in higher molecular weight compounds over the course of the experiment. These experiments could fuel interest in engineering microbial consortia optimized for better waste decomposition during anaerobic digestion. For example, addition of exogenous microorganisms has been shown to enhance decomposition of organic material: both methane production and biodegradation rates increased when experiments were seeded with five strains of aerobic bacteria, which encouraged hydrolysis and acidogenesis in the ‘bioreactor’ environment (Ge et al., 2016).

Understanding the microbial basis for the landfill generation of N_2_O, a more potent GHG gas than CH_4_, has also been a major target of experimentation. With regard to advanced sequencing and transcriptomics methodologies, a possible focus for future work could involve tracking the activity of genes specific to nitrogen cycling processes. A variety of nitrogen cycling genes have been identified both directly from landfill leachate (Zhu et al., 2007) and in experimental approaches. Of particular interest here are the genes associated with nitrification (which produces NO gas) and denitrification (producing N_2_O gas), namely the genes *amoA/pmoA*, *nirKS*, *norBC*, and *nosZ*. A comprehensive study of nitrogen cycling gene activity in landfills has not been conducted. The previously mentioned Likeng and Gouzikeng landfills (e.g., Huang et al., 2002, 2003) hosted a low diversity of *amoA* genes, but substantial and novel diversity of *nosZ* genes in one of the first studies to look for genetic diversity of a gene within landfill materials other than the 16S rRNA gene (Zhu et al., 2007). The impact of evolving modern waste (i.e., an increase in antibiotics or other CECs) is also being considered. For example, it has been found that the addition of the antibiotic sulfamethazine (SMT) to MSW microcosms resulted in a decrease in both NO and NO_2_ (Wu et al., 2017). Effectively, SMT reduced the population of the bacteria carrying the denitrifying genes, *nosZ* and *norB*, and selected for antibiotic resistant strains of *Pseudomonas*, which have fewer denitrifying genes. Wu et al. (2017) also found that adding a different antibiotic, oxytetracycline (OTC), increased the population of taxa that possess *nosZ* and *norB*, increasing the production of NO and N_2_O in these experiments.

Landfill emissions of reduced S gases such as H_2_S can be an odor nuisance and potential health hazard for surrounding communities at some sites. The production of sulfide gases from landfills has been a subject of study for decades (e.g., Fairweather and Barlaz, 1998; Sun and Barlaz, 2015), especially the impact of gypsum wallboard in construction and demolition wastes, high S geological settings, and high sulfate local cover soils. We previously discussed the potential for sulfur-iron transformations in landfill environments, and the close association of sulfate reduction and methanogenesis in various natural environments with evidence that the sulfate and sulfide in landfills can suppress methanogenesis altogether, although results are sparse and require further study (Moreau-Le Golvan et al., 2003). Typically, we consider sulfate reduction to be an anaerobic process, and Long et al. (2016) found that H_2_S production was significantly lower under semi-aerobic conditions than under anaerobic conditions. However, it was also found that the abundance and diversity of sulfate reducing organisms increased nearly 30 times under aerobic conditions (Long et al., 2016). We hypothesize that it is possible that oxygen intrusion stimulates the general degradation of organic material otherwise suppressed by anaerobic conditions, which produces both smaller molecular weight organic compounds and H_2_ stimulating sulfate reducers in localized anaerobic microenvironments. Any H_2_S gas produced could also be re-oxidized, further fueling sulfate reduction. Such speculations would be immensely informed by application of metagenomic and metatranscriptomic methods.

### Elevated Temperature Landfills

Increasing global temperatures will likely impact landfill microbiology, particularly processes in the cover soils. In addition, landfills with internally elevated temperatures, while not common or widespread, are currently being studied as an area of concern (Jafari et al., 2017b). The causes for elevated temperatures in MSW landfills and mechanics of their generation are poorly understood, however, models are being developed and include exothermic pyrolysis of lignocellulosic materials (Barlaz et al., 2017; Benson, 2017; Hao et al., 2017a,b) and considerations of exothermic, abiotic reactions involving aluminum ‘dross’ and selected ash materials in municipal solid wastes (Calder and Stark, 2010; Martin et al., 2013). While very high temperature methanogens are common in many natural environments, elevated temperatures (above 60°C) suppress methanogens in landfills (based on elevated H_2_/CO_2_ in the biogas concurrent with decreased methane). The impact of elevated temperatures on the microbial community and function in landfills is an area of ongoing study, and one which could be improved with application of more advanced and cutting edge technologies.

Both methanogens and methanotrophs in landfills have been shown to be temperature sensitive. Measured gas phase concentrations in ‘hot’ landfills (those above 55°C) show depressed CH_4_, elevated CO_2_ (Benson, 2017), anecdotal evidence of elevated H_2_ (up to 15–20% indicating suppression of methanogenesis; data private domain), and observed CO. Ho and Frenzel (2012) experimentally showed that heat stress (up to 45°C) had a large impact on Type I methanotrophs, and encouraged the growth of Type II methanotrophs, even over that of the control experiment. They postulated that Type II methanotrophs are well suited for stress recovery situations. Advanced sequencing methods could aid in this discussion, by allowing more frequent (even real time) taxonomic analysis or metatranscriptomic analysis to show which organisms are actively consuming methane at given temperature steps. Landfill methanogens have long been known to be sensitive to shifts in temperature and Krakat et al. (2010) found that a shift of even 5°C impacted not only the dominant taxa of their experimental landfill, but also drove the experiments toward hydrogenotrophic methanogenesis. Importantly, the populations of both hydrogenotrophic and acetoclastic methanogens were resilient when temperatures were dropped from 60°C to 55°C, and then increased back to 60°C (Krakat et al., 2010). However, experimentation at higher temperatures under landfill environmental conditions specifically have not been performed.

### Leaking Leachate Problems

While modern landfills in the United States are lined to prevent leachate leakage, many older or poorly designed/managed landfills worldwide contaminate groundwater resources. Early work in tracing leaking leachate plumes (Mouser et al., 2005, 2010) identified that hydrochemical data were not sufficient to track plume fringes, and that profiles of microbial community structure could be used to augment these data to identify changes in plume gradients. Further studies identified the functional diversity of leachate contaminated aquifers, and determined that leachate contamination impacts the diversity, composition, structure, and functional potential of groundwater microbial communities (Lu et al., 2012), and can also be used to pinpoint non contemporaneous point source additions of contaminants from exogenous sources (Prezoisi et al., 2019). Most recently, high throughput sequencing technologies were used to produce metagenomes and metatranscriptomes of a leachate polluted aquifer in the Netherlands (Taş et al., 2018). Taş et al. (2018) reported that the active microbial functions in the contaminated aquifer were involved in the degradation of complex carbon compounds and organic pollutants. For example, genes involved in the catabolism of toluene were more active closer to the sight of contamination (Taş et al., 2018). Together with hydrochemical data, a metatranscriptomic profile could help mitigation strategies by informing whether native populations are actively degrading pollutants, and if not, how to best augment and treat the contaminated groundwater.

## Areas of Potential Future Research

An increased ‘depth’ of sequencing has provided the field of microbial ecology with access to more thorough assessments of microbial diversity, and unlocked the methods of metagenomics and metatranscriptomics as research tools. Single cell genomes of key taxa are less expensive than ever to obtain. With careful experimental design, these tools can be combined with other methodologies (advanced imaging, geochemical and stable isotope analysis, metaproteomics and metalipidomics for example) to ask both targeted and broad questions of landfill microbial ecosystems. Other disciplines related to waste management and contaminant control have already begun to utilize high throughput sequencing technologies in this way (e.g., Sundberg et al., 2013; Miao et al., 2015a, b), and landfill microbiology research will benefit from these examples.

### Using ‘Omics’ Data to Target Landfill Ecosystem Function

Genomic and metagenomic data have been used historically to aid in obtaining difficult to culture, key members of microbial ecosystems. For example, the marine, soil, and extreme microbiology communities have long recognized the power of genome based information in targeting metabolic processes specific to key taxa, toward designing growth media or methods that could better capture them (e.g., Beja et al., 2000a, b; Tyson et al., 2004, 2005; Venter et al., 2004; Assis et al., 2014). Success with using metagenomics and metatranscriptomics to target uncultivated microbial consortia depends on thorough sequence coverage, effective binning, and accurate functional predictions (Lau et al., 2018). Other non-sequencing based methods are also used to determine metabolic function among uncultivated members of microbial communities (e.g., Li et al., 2008; Kinet et al., 2016; Props et al., 2016; Oszust et al., 2018), however, many methodologies such as FISH-SIMS, NanoSIMS, and flow cytometry remain difficult to access for many researchers.

Monitoring of specific landfill ecosystem functions is possible using ‘omics’ based approaches. For example, the activity of methanogens with depth in MSW landfills could be examined using metatranscriptomics or metaproteomics. New genetic markers for methanogenesis have recently been identified, which could allow for the detection of the activity of genes responsible for methanogenesis to be more accurate (Dziewit et al., 2015). Metaproteomics would take this analysis one step further, although such analyses are still technologically and methodologically difficult. However, the data that could be obtained in such approaches could indicate spatially where methane generation is most active – tying this to environmental metadata would be an especially powerful analytical tool to inform MSW management practices. The same concept could be applied to any well known genetic system relevant to landfill ecosystems (e.g., methane oxidation, sulfate reduction, nitrogen cycling, etc.). Experimentally, the microbial response to different treatments could be tracked at the genomic or transcriptomic level, and these data could be co-collected with environmental, proteomic, or lipidomic analyses. Some specific applications are discussed below.

### Impact of Modern Waste Streams

Modern waste streams contain complex mixtures of CECs, reflecting the heterogenous nature of residential (Lehmann, 2015), industrial, and commercial waste (Masoner et al., 2016): United States landfill leachates contain 101 different prescription and non-prescription pharmaceuticals, industrial chemicals, household chemicals, steroid hormones, and animal and plant sterols with concentrations in household and industrial waste at the highest concentrations (∼100–10,000 ng/L). While some studies have investigated the effects of selected CECs, such as pharmaceuticals (e.g., antibiotics; Threedeach et al., 2012; Wang et al., 2015), the impact of CECs on the microbial communities in landfills is largely unknown. Eggen et al. (2010) found both emerging contaminants (such as chlorinated alkylphosphates, pharmaceuticals, and DEET) and legacy contaminants (such as PAHs and phthalates) contaminants at nanogram or microgram per liter concentrations. Antibiotics of different classes affect landfill microbial communities variably, potentially increasing or decreasing the concentration of gases emitted (Wu et al., 2017). A study using next generation sequencing technologies on a Chinese landfill found that landfills may serve as a large reservoir of antibiotic resistant genes with a potentially important role in generating antibiotic resistance (Song et al., 2016). Other modern waste stream compounds of concern include microplastics. Several recent studies have focused on the emergence of microplastics as a concern in the environment (e.g., Kettner et al., 2017; Danso et al., 2019; Jacquin et al., 2019), and one study finds that landfills may be a potential source of microplastics (He et al., 2019). Quantification of microplastics in the environment is still in its infancy, and the lack of standardized methods or regulation makes research in this field difficult to compare. The combination of these findings, in addition to legacy leaking leachate issues, mentioned above, makes it clear that landfills are a potential sink and source for a host of different contaminants that may be detrimental to surrounding environments or human health. Thus further research is needed to understand best management practices for the fate of these contaminants, their effect on adjacent communities, and microbial processes that may assist their mitigation.

### MSW as a Resource

Biogas from landfilled waste as a fuel source has been exploited commercially in the United States since 1975 (Palos Verdes, CA, United States). There were 879 United States landfills with biogas recovery in 2015 (USEPA Greenhouse Gas Reporting Program HH-reporting) and more than 600 sites with commercial biogas utilization, primarily for on-site electrical generation^6^. The Global Methane Initiative^7^ lists 359 international landfill sites with current or planned biogas recovery (see also an older summary by Gendebien et al., 1992). With respect to composting in the United States, Goldstein et al. (2014) report more than 17.6 Tg of garden and food waste annually diverted to more than 4900 composting facilities.

Research in the area of biogas recovery from MSW landfills has often focused on increasing the efficiency of landfilled waste decomposition while balancing the energy/expenses put into the effort, e.g., ‘bioreactor’ landfills discussed above (Ge et al., 2016; Zahedi, 2018; Silas-Moreno et al., 2019). For example, Ge et al. (2016) showed that an addition of exogenous bacteria (i.e., foreign to the landfills), specifically aerobic bacteria, could enhance the hydrolysis and acidogenesis processes of MSW degradation. This resulted in a >63% reduction of volatile solids and a methane production rate of ∼89 L kg^–1^ of organic matter, a threefold increase in methane production over the control experiment. This has economic implications for more precise knowledge of MSW microbial diversity and activity *in situ*.

Advanced sequencing and ‘omics’ based methods are beginning to be applied to these questions; however, studies at present are few, especially with regard to upscaled field applications. In a related field, the hunt for novel and economically useful cellulases has employed metagenomics to reconstruct genomes of microbiota in cow rumen, finding 27,755 putative genes involved in carbohydrate decomposition (Hess et al., 2011). Liu et al. (2015) used metaproteomics to identify the protein producing members of MSW degradation to find main biodegradation pathways. There is also evidence via a metagenomic survey of experimental lignocellulosic microcosms that there are multiple mechanisms of biomass degradation within a landfill microbiome, and that cellulose degrading functionality exists in more taxa than previously known (Ransom-Jones et al., 2017). Because the degradation of cellulosic wastes is of interest both for waste management and biofuel production, advancing technologies is of paramount importance. De Vrieze et al. (2018) argue convincingly that the development of synthetic microbial communities that optimize waste degradation and economic potential is desirable and possible, noting that previous work developed a synthetic community that could produce ethanol with up to 97.7% efficiency (Patle and Lal, 2007; De Vrieze et al., 2018). The generation of ethanol from cellulose waste is typically not economically viable given the current price of fossil fuel resources (e.g., Mandegari et al., 2017a, b; van Rijn et al., 2018), and the discovery and application of organisms and enzymes that catalyze more efficient conversion will be a topic of further research.

### Potential Impacts of Climate Change on Landfill Management and Research

Global climate change can be expected to affect methane emissions from both natural landscapes and engineered soils like landfills (Nazaries et al., 2013) through soil temperature and moisture alterations and feedbacks. For example, older wetland estimates report up to a 78% increase in global CH_4_ emissions if temperatures increased by 3.4°C (Shindell et al., 2004, 2009). Microbial growth rates and efficiency in soils are directly impacted by soil temperature and moisture (e.g., see review by Anthony et al., 2020). The changing global climate can serve as a natural laboratory for understanding how shifts in moisture and temperature affect MSW landfill community composition and the activity of specific groups of landfill microorganisms, both of which will rely on NGS methodology for deeper and more thorough representation. Increasing atmospheric CO_2,_ temperatures, and changes in soil moisture affect a number of soil characteristics and processes, including the relative activities of methanogens and methanotrophs (van Groenigen et al., 2011). It has been postulated that increased atmospheric CO_2_ may increase CH_4_ emissions from soils, either by changing plant respiration rates increasing soil moisture, or by increasing primary productivity, both of which favor methanogenesis over methanotrophy (Singh et al., 2010). Elevated temperatures are expected to increase evaporation and alter rainfall patterns, resulting in increased soil moisture in some regions, which can promote anaerobicity and further favor methanogenesis and the suppression of methanotrophy. Elevated temperature alone can cause a decline in methanotroph abundance in soil (Mohanty et al., 2007). Tracking the activity of relevant genes via metatranscriptomics tied to metagenomics will aid in answering the above questions. However, there is great uncertainty regarding future global CH_4_ emissions: the global effect of elevated temperatures on methanogenesis and methanotrophy is difficult to predict because ecosystem responses will vary with site specific climate, which is an important driver for net seasonal CH_4_ emissions *inclusive* of methanotrophy. For future landfill emissions, regional climate considerations are insufficient because, for specific sites, the combination of site specific cover materials and site specific climate are needed, including variable soil moisture and temperature in each soil over an annual cycle (National Academies of Sciences, Engineering, and Medicine [NASEM], 2018).

Experimental work, both in the laboratory and in the field, followed by NGS aided methodology will be essential in supporting management problems in a changing global climate. In general, previous work and modeling (i.e., Spokas et al., 2015) have suggested that landfill management practices (e.g., cover soil thickness and texture) could be successfully altered to optimize methanotrophy over extended portions of a typical annual cycle in response to temporal changes in soil moisture and temperature. A naturally occurring methanotroph population in landfill cover soils can be diverse enough to recover from environmental changes rapidly. In one recent study, it was shown experimentally that additions of porous adsorbent could improve methane oxidation rates (Han et al., 2016). While management practices do not currently typically involve direct management of cover soil methanotrophs, this practice may become more common in future decades concurrent with changing climate and deeper understanding of temporal populations. In essence tracking the balance of methanotrophy and methanogenesis in MSW landfills in future endeavors will benefit by pairing nucleic acid based research with the landfill gas analyses employed currently, leading to a better understanding of gas sources and sinks, rates, and responsible microorganisms.

## Author Contributions

All authors participated in the literature review and writing of this contribution.

## Conflict of Interest

The authors declare that the research was conducted in the absence of any commercial or financial relationships that could be construed as a potential conflict of interest.

## Footnotes

1 www.epa.gov/lmop

2 https://ec.europa.eu/eurostat/statistics-explained/index.php/Waste_statistics

3 Here and throughout, the term ‘metagenomic’ refers to the sequencing of environmental DNA in a non-targeted approach, to retrieve genomic reads of sequences from multiple organisms in the environmental sample. While the use of the word ‘environmental’ in this context is flexible (e.g., can refer to an experimental sample rather than a sample from a natural environment), the use of the word ‘metagenomic’ is not, and is sometimes incorrectly applied to general 16S rRNA amplicon sequencing. The distinction is important because in the correct usage, it is implied that reads of multiple genes throughout multiple genomes of multiple organisms in the sample have been acquired, perhaps even allowing the construction of ‘MAGs’ (metagenome-assembled genomes). However, in the incorrect application of the term, only one gene (the 16S rRNA gene) is targeted and sequenced, and should more correctly be referred to ‘amplicon sequencing of the 16S rRNA gene.’

4 https://cdn.ymaws.com/www.compostingcouncil.org/resource/resmgr/images/newimagesfolder/Landfill-Ban-Map-2019.png

5 https://www.calrecycle.ca.gov/Nav/Organics

6 www.epa.gov/lmop

7 globalmethane.org

## References

[B1] Andrews W. J. Masoner J. R. Cozzarelli I. M. (2012). Emerging contaminants at a closed and an operating landfill in Oklahoma. *Ground Water Monit. Remediat.* 32:120 10.1111/j.1745-6592.2011.01373.x

[B2] Anthony M. A. Crowther T. W. Maynard D. S. van den Hoogen J. Averill C. (2020). Distinct assembly processes and microbial communities constrain soil organic carbon formation. *One Earth* 2 349–360. 10.1016/j.oneear.2020.03.006

[B3] Asnani P. U. (2006). “Solid waste management,” in *India Infrastructure Report. Urban Infrastructure*, ed. Rastogi A. (New Delhi: Oxford University Press), 186.

[B4] Assis D. A. M. Rezende R. P. Dias J. C. T. (2014). Use of metagenomics and isolation of actinobacteria in Brazil’s Atlantic rainforest soil for antimicrobial prospecting. *ISRN Biotechnol.* 2014:909601. 10.1155/2014/909601 25937991 PMC4393038

[B5] Bareither C. A. Wolfe G. L. McMahon K. D. Benson C. H. (2013). Microbial diversity and dynamics during methane production from municipal solid waste. *Waste Manag.* 33 1982–1992. 10.1016/j.wasman.2012.12.013 23318155

[B6] Barlaz M. A. (1998). Carbon storage during biodegradation of municipal solid waste components in laboratory-scale landfills. *Glob. Biogeochem. Cycles* 12 373–380. 10.1029/98gb00350

[B7] Barlaz M. A. Bareither C. A. Hossain A. Saquing J. Mezzari I. Benson C. H. (2010). Performance of North American bioreactor landfills: II. chemical and biological characteristics. *J. Environ. Eng.* 136 839–853. 10.1061/(asce)ee.1943-7870.0000220 29515898

[B8] Barlaz M. A. Benson C. H. Castaldi M. Luettich S. (2017). Spatial and temporal characteristics of elevated temperatures in municipal solid waste landfills. *Waste Manag.* 59 286–301. 10.1016/j.wasman.2016.10.052 27866996

[B9] Barlaz M. A. Ham R. K. Schaefer D. M. (1989a). Mass-balance analysis of anaerobically decomposed refuse. *J. Environ. Eng.* 115 1088–1102. 10.1061/(asce)0733-9372(1989)115:6(1088)

[B10] Barlaz M. A. Milke M. W. Ham R. K. (1987). Gas production parameters in sanitary landfill simulators. *Waste Manag. Res.* 5 27–39. 10.1016/0734-242x(87)90032-2

[B11] Barlaz M. A. Schaefer D. M. Ham R. K. (1989b). Bacterial population development and chemical characteristics of refuse decomposition in a simulated sanitary landfill. *Appl. Environ. Microbiol.* 55 55–65. 10.1128/aem.55.1.55-65.1989 16347835 PMC184054

[B12] Beja O. Aravind L. Koonin E. V. Suzuki M. T. Hadd A. Nguyen L. P. (2000a). Bacterial rhodopsin: evidence for a new type of phototrophy in the sea. *Science* 289 1902–1906. 10.1126/science.289.5486.1902 10988064

[B13] Beja O. Suzuki M. T. Koonin E. V. Aravind L. Hadd A. Nguyen L. P. (2000b). Construction and analysis of bacterial artificial chromosome libraries from a marine microbial assemblage. *Environ. Microbiol.* 2 516–529. 10.1046/j.1462-2920.2000.00133.x 11233160

[B14] Benson C. (2017). “Characteristics of gas and leachate at an elevated temperature landfill,” in *Geotechnical Frontiers 2017, Waste Containment, Barriers, Remediation, and Sustainable Geoengineering, GSP No. 276*, eds Brandon T. Valentine R. (Geofrontiers: ASCE), 313–322.

[B15] Bodelier P. L. E. Baer-Gilissen M. J. Meima-Franke M. Hordijk K. (2012). Structural and functional response of methane-consuming microbial communities to different flooding regimes in riparian soils. *Ecol. Evol.* 2 106–127. 10.1002/ece3.34 22408730 PMC3297182

[B16] Bogner J. Abdelrafie Ahmed M. Diaz C. Faaij A. Gao Q. Hashimoto S. (2007). “Waste Management,” in *Climate Change 2007: Mitigation. Contribution of Working Group III to the Fourth Assessment Report of the Intergovernmental Panel on Climate Change*, eds Metz B. Davidson O. R. Bosch P. R. Dave R. Meyer L. A. (Cambridge, MA: Cambridge University Press).

[B17] Bogner J. Matthews E. (1999). “Temporal variations in landfill methane emissions: a global perspective,” in *Proceedings Sardinia 99 Seventh International Waste Management and Landfill Symposium, vol. IV, Environmental Impact, Aftercare and Remediation of Landfills*, eds Christensen T. H. Cossu R. Stegmann R. Environ R. (Sardinia: Univ. of Cagliari), 33–42.

[B18] Bogner J. Meadows M. Czepiel P. (1997). Fluxes of methane between landfills and the atmosphere: natural and engineered controls. *Soil Use Manag.* 13 268–277. 10.1111/j.1475-2743.1997.tb00598.x

[B19] Bogner J. Spokas K. Chanton J. (2011). Seasonal greenhouse gas emissions (methane, carbon dioxide, nitrous oxide) from engineered landfills: daily, intermediate, and final California landfill cover soils. *J. Environ. Qual.* 40 1010–1020. 10.2134/jeq2010.0407 21546687

[B20] Bogner J. E. (1990). Controlled study of landfill biodegradation rates using modified BMP assays. *Waste Manag. Res.* 8 329–352. 10.1016/0734-242x(90)90073-v

[B21] Bogner J. E. (1992). Anaerobic burial of refuse in landfills: increased atmospheric methane and implications for increased carbon storage. *Ecol. Bull.* 42 98–108.

[B22] Bogner J. E. Spokas K. A. Burton E. A. (1997). Kinetics of methane oxidation in a landfill cover soil: temporal variations, a whole-landfill oxidation experiment, and modeling of net CH4 emissions. *Environ. Sci. Technol.* 31 2504–2514. 10.1021/es960909a

[B23] Bogner J. E. Sweeney R. Coleman D. Huitric R. Ririe G. T. (1996). Using isotopic and molecular data to model landfill gas processes. *Waste Manag. Res.* 14 367–376. 10.1006/wmre.1996.0037

[B24] Boone D. R. Mah R. A. (1987). “Effect of molecular hydrogen on acetate degradation by *Methanosarcina barkeri*,” in *Perspectives in Biotechnology, NATO ASI Series (Series A: Life Sciences)*, Vol. 128 eds Duarte J. M. C. Archer L. J. Bull A. T. Holt G. (Boston, MA: Springer), 227.

[B25] Börjesson G. Sundh I. Svensson B. (2004). Microbial oxidation of CH4 at different temperatures in landfill cover soils. *FEMS Microb. Ecol.* 48 305–312. 10.1016/j.femsec.2004.02.006 19712300

[B26] Börjesson G. Svensson B. (1997). Nitrous oxide emissions from landfill covers in Sweden, tellus B. *Chem. Phys. Meteorol.* 49 357–363. 10.1034/j.1600-0889.49.issue4.2.x 11841302

[B27] Calder G. V. Stark T. D. (2010). Aluminum reactions and problems in municipal solid waste landfills. *Pract. Period. Hazard. Toxic Radioact. Waste Manag.* 14 258–265. 10.1061/(asce)hz.1944-8376.0000045 29515898

[B28] Castillo-Giménez J. Montañés A. Picazo-Tadeo A. J. (2019). Performance and convergence in municipal waste treatment in the European Union. *Waste Manag.* 85 222–231. 30803576 10.1016/j.wasman.2018.12.025

[B29] Cébron A. Bodrossy L. Chen Y. Singer A. C. Thompson I. P. Prosser J. I. (2007). Identity of active methanotrophs in landfill cover soil as revealed by DNA-stable isotope probing. *FEMS Microbiol. Ecol.* 62 12–23. 10.1111/j.1574-6941.2007.00368.x 17714486

[B30] Chen A.-C. Imachi H. Sekiguchi Y. Ohashi A. Harada H. (2003a). Archaeal community compositions at different depths (up to 30m) of a municipal solid waste landfill in Taiwan as revealed by 16S rDNA cloning analyses. *Biotechnol. Lett.* 25:719. 10.1023/a:1023458631699 12882173

[B31] Chen A.-C. Ueda K. Sekiguchi Y. Ohashi A. Harada H. (2003b). Molecular detection and direct enumeration of methanogenic Archaea and methanotrophic Bacteria in domestic solid waste landfill soils. *Biotechnol. Lett.* 25 1563–1569. 14571983 10.1023/a:1025461915495

[B32] Chen Y. Dumont M. G. Cébron A. Murrell J. C. (2007). Identification of active methanotrophs in a landfill cover soil through detection of expression of 16S rRNA and functional genes. *Environ. Microbiol.* 9:2855. 10.1111/j.1462-2920.2007.01401.x 17922768

[B33] Crespo-Medina M. Twing K. I. Sánchez-Murillo R. Brazelton W. J. McCollom T. M. Schrenk M. O. (2017). Methane dynamics in a tropical serpentinizing environment: the santa elena ophiolite, Costa Rica. *Front. Microbiol.* 8:916. 10.3389/fmicb.2017.00916 28588569 PMC5440473

[B34] Crowther T. W. van den Hoogen J. Wan J. Mayes M. A. Keiser A. D. Mo L. (2019). The global soil community and its influence on biogeochemistry. *Science* 365:eaav0550. 10.1126/science.aav0550 31439761

[B35] Danso D. Chow J. Streit W. R. (2019). Plastics: environmental and biotechnological perspectives on microbial degradation. *Appl. Environ. Microbiol.* 85:e01095-19. 10.1128/AEM.01095-19 PMC6752018 31324632

[B36] De la Cruz F. Chanton J. Barlaz M. (2013). Measurement of carbon storage in landfills from the biogenic carbon content of excavated waste samples. *Waste Manag.* 33 2001–2005. 10.1016/j.wasman.2012.12.012 23332655

[B37] De Vrieze J. Boon N. Verstraete W. (2018). Taking the technical microbiome into the next decade. *Environ. Microbiol.* 20 1991–2000. 10.1111/1462-2920.14269 29745026

[B38] Diaz L. Savage G. Eggerth L. Goluke C. (1996). “Waste management in economically developing countries,” in *Proceedings of the international Solid Waste Assn (Copenhagen) and CALRecovery*, Hercules, CA.

[B39] Donevska K. Peshecski I. Kostadinov T. (2018). “Groundwater pollution threats in the Republic of Macedonia due to uncontrolled landfills,” in *Proceedings of the XVI Danube - European Conference on Geotechnical Engineering* (Hoboken, NJ: Wiley).

[B40] Dumont M. G. Pommerenke B. Casper P. Conrad R. (2011). DNA-, rRNA- and mRNA-based stable isotope probing of aerobic methanotrophs in lake sediment. *Environ. Microbiol.* 13 1153–1167. 10.1111/j.1462-2920.2010.02415.x 21261798

[B41] Dziewit L. Pyzik A. Romaniuk K. Sobczak A. Szczesny P. Lipinski L. (2015). Novel molecular markers for the detection of methanogens and phylogenetic analyses of methanogenic communities. *Front. Microbiol.* 6:694. 10.3389/fmicb.2015.00694 26217325 PMC4493836

[B42] Eggen T. Moeder M. Arukwe A. (2010). Municipal landfill leachates: a significant source for new and emerging pollutants. *Sci. Tot. Environ.* 408 5147–5157. 10.1016/j.scitotenv.2010.07.049 20696466

[B43] Egger M. Rasigraf O. Sapart C. J. Jilbert T. Jetten M. S. M. Röckmann T. (2015). Iron-mediated anaerobic oxidation of methane in brackish coastal sediments. *Environ. Sci. Technol.* 49 277–283. 10.1021/es503663z 25412274

[B44] EMCON (1980). *Associates for Consolidated Concrete Limited, and Alberta Environment. Methane Generation and Recovery from Landfills.* Ann Arbor, MI: Ann Arbor Science Publishers.

[B45] Enning D. Garrelfs J. (2014). Corrosion of iron by sulfate-reducing Bacteria: new views of an old problem. *Appl. Environ. Microbiol.* 80:1226. 10.1128/aem.02848-13 24317078 PMC3911074

[B46] Fairweather R. J. Barlaz M. A. (1998). Hydrogen sulfide production during decomposition of landfill inputs. *J. Environ. Eng.* 124:353 10.1061/(asce)0733-9372(1998)124:4(353)

[B47] Farquhar G. Rovers F. (1973). Gas production during refuse decomposition. *Water Air Soil Pollut.* 2 483–493.

[B48] Fei X. Zekkoa D. Raskin L. (2015). Archaeal community structure in leachate and solid waste is correlated to methane generation and volume reduction during biodegradation of municipal solid waste. *Waste Manag.* 36 184–190. 10.1016/j.wasman.2014.10.027 25481695

[B49] Fisgativa H. Tremier A. Saoudi M. Le Roux S. Dabert P. (2018). Biochemical and microbial changes reveal how aerobic pre-treatment impacts anaerobic biodegradability of food waste. *Waste Manag.* 80 119–129. 10.1016/j.wasman.2018.09.011 30454991

[B50] Ge S. Liu L. Xue Q. Yuan Z. (2016). Effects of exogenous aerobic bacteria on methane production and biodegradation of municipal solid waste in bioreactors. *Waste Manag.* 55:93098. 10.1016/j.wasman.2015.11.024 26601890

[B51] Gebert J. Singh B. K. Pan Y. Bodrossy L. (2009). Activity and structure of methanotrophic communities in landfill cover soils. *Environ. Microbiol. Rep.* 1 414–423. 10.1111/j.1758-2229.2009.00061.x 23765895

[B52] Gendebien A. Pauwels M. Constant M. Lerut-Damanet J. Nyns E. Willumsen H. (1992). *Landfill Gas: From Environment to Energy, Commission of the European Communities.* Final Report EUR 14017/1 EN, Contract 88-B-7030-11-3-17 Brussels: Commission of the European Communities.

[B53] Goldstein N. Coker C. Brown S. (2014). *State of Composting in the U.S.: What, Why, Where, and How.* Washington, DC: Institute for Local Self-Reliance.

[B54] Goodwin S. McPherson J. D. McCombie W. R. (2016). Coming of age: ten years of next-generation sequencing technologies. *Nat. Rev. Genet.* 17:333. 10.1038/nrg.2016.49 27184599 PMC10373632

[B55] Graef C. Hestnes A. G. Svenning M. M. Frenzel P. (2011). The active methanotrophic community in a wetland from the High Arctic. *Environ. Microbiol. Rep.* 3 466–472. 10.1111/j.1758-2229.2010.00237.x 23761309

[B56] Gupta J. Rathour R. Kumar M. Thakur I. S. (2017). Metagenomic analysis of microbial diversity in landfill lysimeter soil of ghazipur landfill site, New Delhi, India. *Genome Announc.* 5 e1104–e1117. 10.1128/genomeA.01104-17 PMC5646401 29051248

[B57] Gurijala K. R. Suflita J. M. (1993). Environmental factors influencing methanogenesis from refuse in landfill samples. *Environ. Sci. Technol.* 27 1176–1181. 10.1021/es00043a018

[B58] Halvadakis C. P. Robertson A. P. Leckie J. O. (1983). *Landfill Methanogenesis: Literature Review and Critique, Final Summary Report.* Stanford University Report 271 Stanford, CA: Dept. of Civil Engineering.

[B59] Hamilton T. L. Peters J. P. Skidmore M. L. Boyd E. S. (2013). Molecular evidence for an active endogenous microbiome beneath glacial ice. *ISME J.* 7 1402–1412. 10.1038/ismej.2013.31 23486249 PMC3695297

[B60] Han J. S. Mahanty B. Yoon S. U. Kim C. G. (2016). Activity of a methanotrophic consortium isolated from landfill cover soil: response to temperature, pH, CO2, and porous adsorbent. *Geomicrobiol. J.* 33:878 10.1080/01490451.2015.1123330

[B61] Hanashima M. Yamasaki K. Kuroki T. Onishi K. (1982). Heat and gas flow analysis in semiaerobic landfill. *J. Environ. Eng. Div. Proc. Am. Soc. Civil Eng.* 107 1–9.

[B62] Hanson J. L. Yesiller N. Oettle N. K. (2010). Spatial and temporal temperature distributions in municipal solid waste landfills. *J. Environ. Eng.* 136 804–814. 10.1061/(ASCE)EE.1943-7870.0000202 29515898

[B63] Hao Z. Sun M. Ducoste J. Barlaz M. (2017a). “IA model to describe heat generation and accumulation at municipal solid waste landfills,” in *Geotechnical Frontiers 2017: Waste Containment, Barriers, Remediation, and Sustainable Geoengineering*, eds Brandon T. L. Valentine R. J. (Washington, DC: ACS Publications), 281–288.

[B64] Hao Z. Sun M. Ducoste J. J. Benson C. H. Luettich S. Castaldi M. J. (2017b). Heat generation and accumulation in municipal solid waste landfills. *Environ. Sci. Technol*. 51 12434–12442. 10.1021/acs.est.7b01844 28933836

[B65] He P. Chen L. Shao L. Zhang H. Lu F. (2019). Municipal solid waste (MSW) landfill: a source of microplastics? -Evidence of microplastics in landfill leachate. *Waste Res.* 159 28–45. 10.1016/j.watres.2019.04.060 31078750

[B66] He P. J. Qu X. Shao L.-M. Li G.-J. Lee D.-J. (2007). Leachate pretreatment for enhancing organic matter conversion in landfill bioreactor. *J. Hazard. Mater.* 142 288–296. 10.1016/j.jhazmat.2006.08.017 16978769

[B67] He R. Wooller M. J. Pohlman J. W. Tiedje J. M. Leigh M. B. (2015). Methane-derived carbon flow through microbial communities in arctic lake sediments. *Environ. Microbiol.* 17:3233. 10.1111/1462-2920.12773 25581131

[B68] Henneberger R. Chiri E. Bodelier P. E. L. Frenzel P. Lüke C. Schroth M. H. (2015). Field-scale tracking of active methane-oxidizing communities in a landfill cover soil reveals spatial and seasonal variability. *Environ. Microbiol.* 17 1721–1737. 10.1111/1462-2920.12617 25186436

[B69] Henneberger R. Lüke C. Mosberger L. Schroth M. H. (2012). Structure and function of methanotrophic communities in a landfill-cover soil. *FEMS Microbiol. Ecol.* 81:52. 10.1111/j.1574-6941.2011.01278.x 22172054

[B70] Herrera L. K. Videla H. A. (2009). Role of iron-reducing bacteria in corrosion and protection of carbon steel. *Int. Biodeteriorat. Biodegradat.* 63 891–895. 10.1016/j.ibiod.2009.06.003

[B71] Héry M. Singer A. C. Kumaresan D. Bodrossy L. Stralis-Pavese N. Prosser J. I. (2008). Effect of earthworms on the community structure of active methanotrophic bacteria in a landfill cover soil. *ISME J.* 2 92–104. 10.1038/ismej.2007.66 18049457

[B72] Hess M. Sczyrba A. Egan R. Kim T. W. Chokhawala H. Schroth G. (2011). Metagenomic discovery of biomass-degrading genes and genomes from cow rumen. *Science* 331 463–467. 10.1126/science.1200387 21273488

[B73] Ho A. Frenzel P. (2012). Heat stress and methane-oxidizing bacteria: effects on activity and population dynamics. *Soil Biol. Biochem.* 50 22–25. 10.1016/j.soilbio.2012.02.023

[B74] Ho A. Lüke C. Frenzel P. (2011). Recovery of methanotrophs from disturbance: population dynamics, evenness and functioning. *ISME J.* 5 750–758. 10.1038/ismej.2010.163 20981115 PMC3105735

[B75] Ho A. van den Brink E. Reim A. Krause S. M. B. Bodelier P. L. E. (2016). Recurrence and frequency of disturbance have cumulative effect on methanotrophic activity, abundance, and community structure. *Front. Microbiol.* 6:1493. 10.3389/fmicb.2015.01493 26779148 PMC4700171

[B76] Hoornweg D. Bhada-Tata P. (2012). *What a Waste: A Global Review of Solid Waste Management.* Washington, DC: World Bank.

[B77] Hoshino T. Inagaki F. (2017). Distribution of anaerobic carbon monoxide dehydrogenase genes in deep subseafloor sediments. *Lett. Appl. Microbiol.* 64 355–363. 10.1111/lam.12727 28256106

[B78] Huang L.-N. Chen Y.-Q. Zhou H. Luo S. Lan C.-Y. Qu L.-H. (2003). Characterization of methanogenic *Archaea* in the leachate of a closed municipal solid waste landfill. *FEMS Microbiol. Ecol.* 46 171–177. 10.1016/s0168-6496(03)00218-6 19719570

[B79] Huang L.-N. Zhou H. Zhu S. Qu L.-H. (2004). Phylogenetic diversity of bacteria in the leachate of a full-scale recirculating landfill. *FEMS Microbiol. Ecol.* 50 175–183. 10.1016/j.femsec.2004.06.008 19712358

[B80] Huang L.-N. Zhoum H. Chen Y.-Q. Luo S. Lan C.-Y. Qu L.-H. (2002). Diversity and structure of the archaeal community in the leachate of a full-scale recirculating landfill as examined by direct 16S rRNA gene sequence retrieval. *FEMS Microbiol. Lett.* 214 235–240. 10.1111/j.1574-6968.2002.tb11353.x 12351237

[B81] Huang L.-N. Zhu S. Zhou H. Qu L.-H. (2005). Molecular phylogenetic diversity of bacteria associated with the leachate of a closed municipal solid waste landfill. *FEMS Microbiol. Lett.* 242 297–303. 10.1016/j.femsle.2004.11.021 15621451

[B82] Huber-Humer M. (2004). *Abatement of Landfill Methane Emissions by Microbial Oxidation in Biocovers Made of Compost.* Ph.D. thesis, University of Natural Resources and Applied Life Sciences, Vienna.

[B83] Hug L. A. Baker B. J. Anantharaman K. Brown C. T. Probst A. J. Castelle C. (2016). A new view of the tree of life. *Nat. Microbiol.* 1:16048. 10.1038/NMICROBIOL.2016.48 27572647

[B84] IPCC (1996). *Guidelines for National Greenhouse Gas Inventories.* Geneva: IPCC.

[B85] IPCC (2006). *Guidelines for National Greenhouse Gas Inventories.* Hayama: IPCC.

[B86] Jacquin J. Cheng J. Odobel C. Pandin C. Conan P. Pujo-Pay M. (2019). Microbial ecotoxicology of marine plastic debris: a review on colonization and biodegradation by the “Plastisphere”. *Front. Microbiol.* 10:865. 10.3389/fmicb.2019.00865 31073297 PMC6497127

[B87] Jafari N. H. Stark T. D. Thalhamer T. (2017a). Progression of elevated temperatures in municipal solid waste landfills. *J. ASCE Geotechn. Geoeng. Div.* 143:05017004. 10.1061/(ASCE)GT.1943-5606.0001683 29515898

[B88] Jafari N. H. Stark T. D. Thalhamer T. (2017b). Spatial and temporal characteristics of elevated temperatures in municipal solid waste landfills. *Waste Management.* 59 286–301. 10.1016/j.wasman.2016.10.052 27866996

[B89] Jansson J. K. Taş N. (2014). The microbial ecology of permafrost. *Nat. Rev. Microbiol.* 12 414–425. 10.1038/nrmicro3262 24814065

[B90] José León M. Fernández A. B. Ghai R. Sánchez-Porro C. Rodriguez-Valera F. Ventosa A. (2014). From metagenomics to pure culture: isolation and characterization of the moderately halophilic bacterium *Spiribacter salinus* gen. nov., sp. nov. *Appl. Environ. Microbiol.* 80:3850. 10.1128/aem.00430-14 24747894 PMC4054224

[B91] Juottonen H. Galand P. E. Tuittila E. S. (2005). Methanogen communities and Bacteria along an ecohydrological gradient in a northern raised bog complex. *Environ. Microbiol.* 7 1547–1557. 10.1111/j.1462-2920.2005.00838.x 16156728

[B92] Kasali G. B. Senior E. Watson-Craik I. A. (1988). Preliminary investigation of the influence of pH on the solid-state refuse methanogenic fermentation. *J. Appl. Bacteriol.* 65 231–239. 10.1111/j.1365-2672.1988.tb01890.x

[B93] Kettner M. T. Rojas-Jimenez K. Oberbeckmann S. Labrenz M. Grossart H.-P. (2017). Microplastics alter composition of fungal communities in aquatic ecosystems. *Environ. Microbiol.* 19 4447–4459. 10.1111/1462-2920.13891 28805294

[B94] Kinet R. Dzaomuho P. Baert J. Taminiau B. Daube G. Nezer C. (2016). Flow cytometry community fingerprinting and amplicon sequencing for the assessment of landfill leachate cellulolytic bioaugmentation. *Bioresour. Technol.* 214 450–459. 10.1016/j.biortech.2016.04.131 27160955

[B95] Kip N. Ouyang W. van Winden J. Raghowbarsing A. van Niftrik L. Pol A. (2011). Detection, isolation, and characterization of acidophilic methanotrophs from sphagnum mosses. *Appl. Environ. Microbiol.* 77:5643. 10.1128/AEM.05017-11 21724892 PMC3165258

[B96] Kip N. van Veen J. A. (2015). The dual role of microbes in corrosion. *ISME J.* 9:542. 10.1038/ismej.2014.169 25259571 PMC4331587

[B97] Kjeldsen P. Barlaz M. A. Rooker A. P. Baun A. Ledin A. Christensen T. H. (2002). Present and long-term composition of MSW landfill leachate: a review. *Crit. Rev. Environ. Sci. Technol.* 32 297–336. 10.1080/10643380290813462

[B98] Köchling T. Sanz J. L. Gavazza S. Florencio L. (2015). Analysis of microbial community structure and composition in leachates from a young landfill by 454 pyrosequencing. *Appl. Microbiol. Biotechnol.* 99 5657–5668. 10.1007/s00253-015-6409-4 25652654

[B99] Kolb S. Horn M. A. (2012). Microbial CH4 and N2O consumption in acidic wetlands. *Front. Microbiol.* 3:78. 10.3389/fmicb.2012.00078 22403579 PMC3291872

[B100] Krakat N. Westphal A. Schmidt S. Scherer P. (2010). Anaerobic digestion of renewable biomass: thermophilic temperature governs methanogen population dynamics. *Appl. Environ. Microbiol.* 76 1842–1850. 10.1128/aem.02397-09 20097828 PMC2838025

[B101] Krause S. Lüke C. Frenzel P. (2012). Methane source strength and energy flow shape methanotrophic communities in oxygen–methane counter-gradients. *Environ. Microbiol. Rep.* 4 203–208. 10.1111/j.1758-2229.2011.00322.x 23757274

[B102] Krishnamurthi S. Chakrabarti T. (2013). Diversity of Bacteria and Archaea from a landfill in Chandigarh, Indai as revealed by culture-dependent and culture-independent molecular approaches. *Syst. Appl. Microbiol.* 36 56–68. 10.1016/j.syapm.2012.08.009 23274043

[B103] Kwon M. J. O’Loughlin E. J. Boyanov M. I. Brulc J. M. Johnston E. R. Kemner K. M. (2016). Impact of organic carbon electron donors on microbial community development under iron- and sulfate-reducing conditions. *PLoS One* 11:e0146689. 10.1371/journal.pone.0146689 26800443 PMC4723079

[B104] Ladapo J. A. Barlaz M. A. (1997). Isolation and characterization of refuse methanogens. *J. Appl. Microbiol.* 82 751–758. 10.1046/j.1365-2672.1997.00154.x

[B105] Laloui-Carpentier W. Li T. Vigneron V. Mazéas L. Bouchez T. (2006). Methanogenic diversity and activity in municipal solid waste landfill leachates. *Antonie Van Leeuwenhoek* 89 423–434. 10.1007/s10482-005-9051-9 16779637

[B106] Lau M. Cameron C. Magnabosco C. Brown C. T. Schilkey F. Grim S. (2014). Phylogeny and phylogeography of functional genes shared among seven terrestrial subsurface metagenomes reveal N-cycling and microbial evolutionary relationships. *Front. Microbiol.* 5:531. 10.3389/fmicb.2014.00531 25400621 PMC4215791

[B107] Lau M. C. Y. Harris R. L. Oh Y. Yi M. J. Behmard A. Onstott T. C. (2018). Taxonomic and functional compositions impacted by the quality of metatranscriptomic assemblies. *Front. Microbiol.* 9:1235 10.3389/fmicb.2018.01235 PMC6019464 29973918

[B108] Lehmann L. (2015). The garbage project revisited: from a 20th century archaeology of food waste to a contemporary study of food packaging waste. *Sustainability* 7 6994–7010. 10.3390/su7066994

[B109] Li S. Zhang Y. Liu J. Yu M. (2008). Corrosion behavior of steel A3 influenced by *Thiobacillus ferrooxidans*. *Acta Physico Chim. Sin.* 24 1553–1557. 10.1016/s1872-1508(08)60063-7

[B110] Liebner S. Rublack K. Stuehrmann T. Wagner D. (2009). Diversity of aerobic methanotrophic bacteria in a permafrost active layer soil of the Lena Delta, Siberia. *Microbiol. Ecol.* 57 25–35. 10.1007/s00248-008-9411-x 18592300

[B111] Lin B. Monreal C. M. Tambong J. T. Miguez C. B. Carrasco-Medina L. (2009). Phylogenetic analysis of methanotrophic communities in cover soils of a landfill in Ontario. *Can. J. Microbiol.* 55 1103–1112. 10.1139/w09-069 19898553

[B112] Liu D. Li M. Xi B. Zhao Y. Wei Z. Song C. (2015). Metaproteomics reveals major microbial players and their biodegradation functions in a large-scale aerobic composting plant. *Microb. Biotechnol.* 8 950–960. 10.1111/1751-7915.12290 25989417 PMC4621448

[B113] Long Y. Fang Y. Shen D. Feng H. Chen T. (2016). Hydrogen sulfide (H2S) emission control by aerobic sulfate reduction in landfill. *Nat. Sci. Rep.* 6:38103. 10.1038/srep38103 27909309 PMC5133566

[B114] Lu Z. He Z. Parisi V. A. Kang S. Deng Y. Van Nostrand J. D. (2012). Geochip-based analysis of microbial functional gene diversity in a landfill leachate-contaminated aquifer. *Environ. Sci. Technol.* 46 5824–5833. 10.1021/es300478j 22533634

[B115] Maliva R. G. Missimer T. M. Leo K. C. Statom R. A. Dupraz C. Lynn M. (2000). Unusual calcite stromatolites and pisoids from a landfill leachate collection system. *Geology* 28 931–934. 10.1130/0091-7613(2000)028<0931:ucsapf>2.3.co;2

[B116] Mandegari M. A. Farzad S. Görgens J. F. (2017a). Economic and environmental assessment of cellulosic ethanol production scenarios annexed to a typical sugar mill. *Bioresour. Technol.* 224 314–326. 10.1016/j.biortech.2016.10.074 27816352

[B117] Mandegari M. A. Farzad S. van Rensburg E. Görgens J. F. (2017b). Multi-criteria analysis of a biorefinery for co-production of lactic acid and ethanol from sugarcane lignocellulose. *Biofuels Bioprod. Bioref.* 11 971–990. 10.1002/bbb.1801

[B118] Mantere T. Kersten S. Hoischen A. (2019). Long-read sequencing emerging in medical genetics. *Front. Genet.* 10:426. 10.3389/fgene.2019.00426 31134132 PMC6514244

[B119] Marlow J. J. Steele J. A. Case D. H. Connon S. A. Levin L. A. Orphan V. J. (2014). Microbial abundance and diversity patterns associated with sediments and carbonates from the methane seep environments of Hydrate Ridge, OR. *Front. Mar. Sci.* 1:44 10.3389/fmars.2014.00044

[B120] Martin J. W. Stark T. D. Thalhamer T. Gerbasi-Graf G. T. Gortner R. E. (2013). Detection of aluminum waste reactions and waste fires. *J. Hazard. Toxic Radioact. Waste* 17 164–174. 10.1061/(asce)hz.2153-5515.0000171 29515898

[B121] Masoner J. R. Kolpin D. W. Furlong E. T. Cozzarelli I. M. Gray J. L. (2016). Landfill leachate as a mirror of today’s disposable society: pharmaceuticals and other contaminants of emerging concern in final leachate from landfills in the conterminous United States. *Environ. Toxicol. Chem.* 35:906. 10.1002/etc.3219 26562222

[B122] Masoner J. R. Kolpin D. W. Furlong E. T. Cozzarelli I. M. Gray J. L. Schwab E. A. (2014). Contaminants of emerging concern in fresh leachate from landfills in the conterminous United States. *Environ. Sci. Process. Impacts* 16 2335–2354. 10.1039/c4em00124a 25111596

[B123] Medina M. (2007). *The World’s Scavengers: Salvaging for Sustainable Production and Consumption.* Thousand Oaks, CA: Altamira Press.

[B124] Miao Y. Liao R. Zhang X.-X. Liu B. Li Y. Wu B. (2015a). Metagenomic insights into salinity effect on diversity and abundance of denitrifying bacteria and genes in an expanded granular sludge bed reactor treating high-nitrate wastewater. *Chem. Eng. J.* 277 116–123. 10.1016/j.cej.2015.04.125

[B125] Miao Y. Liao R. Zhang X.-X. Wang Y. Wang Z. Shi P. (2015b). Metagenomic insights into Cr(VI) effect on microbial communities and functional genes of an expanded granular sludge bed reactor treating high-nitrate wastewater. *Water Res.* 76 43–52. 10.1016/j.watres.2015.02.042 25792433

[B126] Milucka J. Ferdelman T. G. Polerecky L. Franzke D. Wegener G. Schmid M. (2012). Zero-valent sulphur is a key intermediate in marine methane oxidation. *Nature* 491 541–546. 10.1038/nature11656 23135396

[B127] Mohanty S. R. Bodelier P. L. Conrad R. (2007). Effect of temperature on composition of the methanotrophic com- munity in rice field and forest soil. *FEMS Microbiol. Ecol.* 62 24–31. 10.1111/j.1574-6941.2007.00370.x 17725622

[B128] Momper L. Reese B. K. Zinke L. Wanger G. Osburn M. R. Moser D. (2017). Major phylum-level differences between porefluid and host rock bacterial communities in the terrestrial dep subsurface. *Environ. Microbiol. Rep.* 9 501–511. 10.1111/1758-2229.12563 28677247

[B129] Moreau-Le Golvan Y. Triquinaux C. Bogner J. Smith L. Munoz M. (2003). “Methanogenesis optimisation by controlling ph and sulfate in applied research for woodlawn bioreactor [Australia],” in *Proceedings Sardinia 9th International Landfill Research Symposium* (Caligari: CISA).

[B130] Mormile M. R. Gurijala K. R. Robinson J. A. McInerney M. J. Suflita J. M. (1996). The importance of hydrogen in landfill fermentations. *Appl. Environ. Microbiol.* 62 1583–1588. 10.1128/aem.62.5.1583-1588.1996 16535310 PMC1388848

[B131] Mouser P. J. Rizzo D. M. Druschel G. K. Morales S. E. Hayden N. O’Grady P. (2010). Enhanced detection of groundwater contamination from a leaking waste disposal site by microbial community profiles. *Water Resour. Res.* 46:W12506 10.1029/2010WR009459

[B132] Mouser P. J. Rizzo D. M. Röling F. M. Van Breukelen B. M. (2005). A multivariate statistical approach to spatial representation of groundwater contamination using hydrochemistry and microbial community profiles. *Environ Sci Technol.* 39 7551–7559. 10.1021/es0502627 16245827

[B133] Muaaz-Us-Salam S. Cleall P. J. Harbottle M. J. (2019). The case for examining fluid flow in municipal solid waste at the pore-scale – A review. *Waste Manag. Res.* 37 315–332. 10.1177/0734242x19828120 30791834

[B134] Mukred A. M. Hamid A. A. Hamzah A. Yusoff W. M. W. (2008). Development of three bacteria consortium for the bioremediation of crude petroleum-oil in contaminated water. *Online J. Biol. Sci.* 8 73–79. 10.3844/ojbsci.2008.73.79

[B135] Nair I. Zhang X. Gutierrez J. Chen J. Egolfopoulos F. Tsotsis T. (2013). Effect of siloxanes contained in natural gas on the operation of a residential furnace. *Ind. Eng. Chem. Res.* 52 6253–6261. 10.1021/ie400449y

[B136] National Academies of Sciences, Engineering, and Medicine [NASEM] (2018). *Improving Characterization of Anthropogenic Methane Emissions in the United States.* Washington, DC: The National Academies Press. 30110140

[B137] Nazaries L. Murrell J. C. Millard P. Baggs L. Singh B. K. (2013). Methane, microbes, and models: fundamental understanding of the soil methane cycle for future predictions. *Environ. Microbiol.* 15 2395–2417. 10.1111/1462-2920.12149 23718889

[B138] Nobu M. K. Narihiro T. Rinke C. Kamagata Y. Tringe S. G. Woyke T. (2015). Microbial dark matter ecogenomics reveals complex synergistic networks in a methanogenic bioreactor. *ISME J.* 9 1710–1722. 10.1038/ismej.2014.256 25615435 PMC4511927

[B139] Ojuri O. O. Ayodele F. O. Oluwatuyi O. E. (2018). Risk assessment and rehabilitation potential of a millennium city dumpsite in Sub-Saharan Africa. *Waste Manag.* 76 621–628. 10.1016/j.wasman.2018.03.002 29548830

[B140] Oonk H. Boom T. (1995). Validation of landfill gas formation models. *Stud. Environ. Sci.* 65 597–602. 10.1016/s0166-1116(06)80251-7

[B141] Osburn M. R. LaRowe D. E. Momper L. M. Amend J. P. (2014). Chemolithotrophy in the continental deep subsurface: sanford underground research facility (SURF), USA. *Front. Microbiol.* 5:610. 10.3389/fmicb.2014.00610 25429287 PMC4228859

[B142] Oszust K. Gryta A. Ziemiński K. Bilińska-Wielgus N. Gałązka R. Frąc M. (2018). Characterization of microbial functional and genetic diversity as a novel strategy of biowaste ecotoxicological evaluation. *Int. J. Environ. Sci. Technol.* 16 4261–4274. 10.1007/s13762-018-2066-3

[B143] Patel A. Belykh E. Miller E. J. George L. L. Martirosyan N. L. Byvaltsev V. A. (2018). MinION rapid sequencing: review of potential applications in neurosurgery. *Surg. Neurol. Int.* 9:157 10.4103/sni.sni_55_18 PMC6094492 30159201

[B144] Patle S. Lal B. (2007). Ethanol production from hydrolysed agricultural wastes using mixed culture of *Zymomonas mobilis* and *Candida tropicalis*. *Biotechnol. Lett.* 29 1839–1843. 10.1007/s10529-007-9493-4 17657407

[B145] Pohland F. G. (1980). Leachate recycle as landfill management option. *J. Environ. Eng.* 106 1057–1069.

[B146] Powell J. Townsend T. Zimmerman J. (2016). Estimates of solid waste disposal rates and reduction targets for landfill gas emissions. *Nat. Clim. Change Lett.* 6 162–165. 10.1038/NCLIMATE2804

[B147] Prezoisi E. Frollini E. Zoppini A. Ghergo S. Melita M. Parrone D. (2019). Disentangling natural and anthropogenic impacts on groundwater by hydrogeochemical, isotopic and microbiological data: hints from a municipal solid waste landfill. *Waste Manag.* 84 245–255. 10.1016/j.wasman.2018.12.005 30691899

[B148] Props R. Monsieurs P. Mysara M. Clement L. Boon N. (2016). Measuring the biodiversity of microbial communities by flow cytometry. *Methods Ecol. Evol.* 7 1376–1385. 10.1111/2041-210x.12607

[B149] Qu X. Mazéas L. Vavilin V. A. Epissard J. Lemunier M. Mouchel J.-M. (2009). Combined monitoring of changes in δ13CH4 and archaeal community structure during mesophilic methanization of municipal solid waste. *FEMS Microbiol. Ecol.* 68 236–245. 10.1111/j.1574-6941.2009.00661.x 19302549

[B150] Rajasekar A. Maruthamuthu S. Muthukumar N. Mohanan S. Subramanian P. Palaniswamy N. (2005). Bacterial degradation of naphtha and its influence on corrosion. *Corros. Sci.* 47 257–271. 10.1016/j.corsci.2004.05.016

[B151] Ransom-Jones E. McCarthy A. J. Haldenby S. Doonan J. McDonald J. E. (2017). Lignocellulose-degrading microbial communities in landfill sites represent a repository of unexplored biomass-degrading diversity. *Appl. Environ. Sci.* 2 e300–e317. 10.1128/mSphere.00300-17 PMC5541161 28776044

[B152] Rathje W. Murphy C. (1992). *RUBBISH! The Archaeology of Garbage.* New York, NY: HarperCollins Publishers.

[B153] Reinhart D. Townsend T. (1997). *Landfill Bioreactor: Design and Operation.* Boca Raton, FL: Lewis Publishers.

[B154] Reinhart D. R. McCreanor P. T. Townsend T. (2002). The bioreactor landfill: its status and future. *Waste Manag. Resour.* 20 172–186. 10.1177/0734242x0202000209 12058823

[B155] Ritzkowski M. Stegmann R. (2012). Landfill aeration worldwide: concepts, indications and findings. *Waste Manag.* 32 1411–1419. 10.1016/j.wasman.2012.02.020 22459512

[B156] Robb F. T. Techtmann S. M. (2018). Life on the fringe: microbial adaptation to growth on carbon monoxide. *F1000Research* 7:1981. 10.12688/f1000research.16059.1 30647903 PMC6317499

[B157] Scheutz C. Kjeldsen P. Bogner J. De Visscher A. Gebert J. Hilger H. (2009). Microbial methane oxidation processes and technologies for mitigation of landfill gas emissions. *Waste Manag. Res*. 27 409–455. 10.1177/0734242X09339325 19584243

[B158] Serrano-Silva N. Sarria-Guzman Y. Dendooven L. Luna-Guido M. (2014). Methanogenesis and methanotrophy in soil: a review. *Pedosphere* 24 291–317.

[B159] Shen L. Ouyang L. Zhu Y. Trimmer M. (2019). Active pathways of anaerobic methane oxidation across contrasting riverbeds. *ISME J.* 13 752–766. 10.1038/s41396-018-0302-y 30375505 PMC6461903

[B160] Shindell D. T. Faluvegi G. Koch D. M. Schmidt G. A. Unger N. Bauer S. E. (2009). Improved attribution of climate forcing to emissions. *Science* 326 716–718. 10.1126/science.1174760 19900930

[B161] Shindell D. T. Walter B. P. Faluvegi G. (2004). Impacts of climate change on methane emissions from wetlands. *Geophys. Res. Lett.* 31:L21202.

[B162] Shrestha M. Shrestha P. M. Frenzel P. Conrad R. (2010). Effect of nitrogen fertilization on methane oxidation, abundance, community structure, and gene expression of methanotrophs in the rice rhizosphere. *ISME J.* 4 1545–1556. 10.1038/ismej.2010.89 20596069

[B163] Silas-Moreno M. V. Senés-Guerrero C. Pacheco A. Montesinos-Castellanos A. (2019). Methane potential and metagenomics of wastewater sludge and a methane-producing landfill solid sample as microbial inocula for anaerobic digestion of food waste. *J. Chem. Technol. Biotechnol.* 94 1123–1133. 10.1002/jctb.5859

[B164] Singh B. K. Bardgett R. D. Smith P. Reay D. S. (2010). Microorganisms and climate change: terrestrial feedbacks and mitigation options. *Nat. Rev. Microbiol.* 8 779–790. 10.1038/nrmicro2439 20948551

[B165] Sivan O. Antler G. Turchyn A. V. Marlow J. J. Orphan V. J. (2014). Iron oxides stimulate sulfate-driven anaerobic methane oxidation in seeps. *Proc. Natl. Acad. Sci. U.S.A.* 111 E4139–E4147. 25246590 10.1073/pnas.1412269111 PMC4209987

[B166] Slezak R. Krzystek L. Ledakowicz S. (2015). Degradation of municipal solid waste in simulated landfill bioreactors under aerobic conditions. *Waste Manag.* 43 293–299. 10.1016/j.wasman.2015.06.017 26119011

[B167] Song L. Li L. Yang S. Lan J. He H. McElmurry S. P. (2016). Sulfamethoxazole, tetracycline and oxytetracycline and related antibiotic resistance genes in a large-scale landfill, China. *Sci. Total Environ.* 551 9–15. 10.1016/j.scitotenv.2016.02.007 26874755

[B168] Song L. Wang Y. Tang W. Lei Y. (2015). Archaeal community diversity in municipal waste landfill sites. *Appl. Microb. Biotechnol.* 99 6125–6137. 10.1007/s00253-015-6493-5 25758957

[B169] Spokas K. Bogner J. (2011). Limits and dynamics of methane oxidation in landfill cover soils. *Waste Manag.* 31 823–832. 10.1016/j.wasman.2009.12.018 20096554

[B170] Spokas K. Bogner J. Chanton J. (2011). A process-based inventory model for landfill CH4 emissions inclusive of soil microclimate and seasonal methane oxidation. *J. Geophys. Res. Biogeosci.* 116:G04017.

[B171] Spokas K. Bogner J. Chanton J. Morcet M. Aran C. Graff C. (2006). Methane mass balance at three landfill sites: what is the efficiency of capture by gas collection systems? *Waste Manag.* 26 516–525. 10.1016/j.wasman.2005.07.021 16198554

[B172] Spokas K. Bogner J. Corcoran M. Walker S. (2015). From California dreaming to California data: challenging historic models for landfill CH4 emissions. *Elementa* 3:000051 10.12952/journal.elementa.000051

[B173] Staley B. F. de los Reyes F. L. III Barlaz M. A. (2012). Comparison of Bacteria and Archaea communities in municipal solid waste, individual refuse components, and leachate. *FEMS Microbiol. Ecol.* 79 465–473. 10.1111/j.1574-6941.2011.01239.x 22092358

[B174] Staley B. F. de los Reyes F. L. III Wang L. Barlaz M. A. (2018). Microbial ecological succession during municipal solid waste decomposition. *Appl. Microbiol. Biotechnol.* 102 5731–5740. 10.1007/s00253-018-9014-5 29705953

[B175] Staley B. F. Saikaly P. E. de los Reyes F. L. Barlaz M. A. (2011). Critical evaluation of solid waste sample processing for DNA-based microbial community analysis. *Biodegradation* 22 189–204. 10.1007/s10532-010-9387-3 20652623

[B176] Stamps B. W. Lyles C. N. Sulflita J. M. Masoner J. R. Cozzarelli I. M. Kolpin D. W. (2016). Municipal solid waste landfills harbor distinct microbiomes. *Front. Microbiol.* 7:534. 10.3389/fmicb.2016.00534 27148222 PMC4837139

[B177] Stephens L. Fuller D. Bolvin N. (2019). Archaeological assessment reveals earth’s early transformation through land use. *Science* 365 897–902. 10.1126/science.aax1192 31467217

[B178] Stralis-Pavese N. Bodrossy L. Reichenauer T. G. Weilharter A. Sessitsch A. (2006). 16S rRNA based T-RFLP analysis of methane oxidising bacteria – assessment, critical evaluation of methodology performance and application for landfill site cover soils. *Appl. Soil Ecol.* 31 251–266. 10.1016/j.apsoil.2005.05.006

[B179] Strasser S. (1999). *Waste and Want: A Social History of Trash.* New York, NY: Henry Holt and Company.

[B180] Sun W. Barlaz M. A. (2015). Measurement of chemical leaching potential of sulfate from landfill disposed sulfate containing wastes. *Waste Manag.* 36:191. 10.1016/j.wasman.2014.11.014 25499684

[B181] Sundberg C. Al-Soud W. A. Larsson M. Alm E. Yekta S. S. Svensson B. H. (2013). 454 pyrosequencing analyses of bacterial and archaeal richness in 21 full-scale biogas digesters. *FEMS Microbiol. Ecol.* 85 612–623. 23678985 10.1111/1574-6941.12148

[B182] Tajima K. Aminov R. I. Nagamine T. Ogata K. Nakamura M. Matsui H. (1999). Rumen bacterial diversity as determined by sequence analysis of 16S rDNA libraries. *FEMS Microbiol. Ecol.* 29 159–169. 10.1111/j.1574-6941.1999.tb00607.x

[B183] Tang W. Wang Y. Lei Y. Song L. (2016). Methanogen communities in a municipal landfill complex in China. *FEMS Microbiol. Lett.* 363:fnw075. 10.1093/femsle/fnw075 27036145

[B184] Taş N. Brandt B. W. Brater M. van Breukelen B. M. Röling W. F. M. (2018). Subsurface landfill leachate contamination affects microbial metabolic potential and gene expression in the *Banisveld aquifer*. *FEMS Microbiol. Ecol.* 94:fiy156. 10.1093/femsec/fiy156 30124822

[B185] Threedeach S. Chiemchairsi W. Watanabe T. Chiemchaisri C. Honda R. Yamamoto K. (2012). Antibiotic resistance of Escherichia coli in leachates from municipal solid waste landfills: comparison between semi-aerobic and anaerobic operations. *Bioresour. Technol.* 113 253–258. 10.1016/j.biortech.2012.01.086 22330590

[B186] Tissot B. P. Welte D. H. (1984). *Petroleum Formation and Occurrence*. 2nd Edn Berlin: Springer-Verlag, 699.

[B187] Tyson G. W. Chapman J. Hugenholtz P. Allen E. E. Ram R. J. Richardson P. M. (2004). Community structure and metabolism through reconstruction of microbial genomes from the environment. *Nature* 428 37–43. 10.1038/nature02340 14961025

[B188] Tyson G. W. Lo I. Baker B. J. Allen E. E. Hugenholtz P. Banfield J. F. (2005). Genome-directed isolation of the key nitrogen fixer *Leptospirillum ferrodiazotrophum* sp. nov. from an acidophilic microbial community. *Appl. Environ. Microbiol.* 71:6319. 10.1128/aem.71.10.6319-6324.2005 16204553 PMC1266007

[B189] Uz I. Rasche M. E. Townsend T. Ogram A. V. Lindner A. S. (2003). Characterization of methanogenic and methanotrophic assemblages in landfill samples. *Proc. R. Soc. Lond. B* 270 S202–S205. 10.1098/rsbl.2003.0061 PMC1809964 14667383

[B190] van Groenigen K. J. Osenberg C. W. Hungate B. A. (2011). Increased soil emissions of potent greenhouse gases under increased atmospheric CO2. *Nature* 475 214–216. 10.1038/nature10176 21753852

[B191] Van Haaren R. Themelis N. Goldstein N. (2010). State of garbage in America. *Biocycle* 51:16.

[B192] van Rijn R. Nieves I. U. Shanmugman K. T. Ingram L. O. Vermerris W. (2018). Techno-economic evaluation of cellulosic ethanol production based on pilot biorefinery data: a case study of sweet sorghum bagasse processed via L+SScF. *BioEnergy Res.* 11 414–425. 10.1007/s12155-018-9906-3

[B193] Vavilin V. A. Jonsson S. Ejlertsson J. Swenson B. H. (2006). Modelling MSW decomposition under landfill conditions considering hydrolytic and methanogenic inhibition. *Biodegradation* 17 389–402. 10.1007/s10532-005-9009-7 16477363

[B194] Venter J. C. Remington K. Heidelberg J. F. (2004). Environmental genome shotgun sequencing of the Sargasso Sea. *Science* 304 66–74. 10.1126/science.1093857 15001713

[B195] Vigneron A. Alsop E. B. Cruaud P. Philibert G. King B. Leslie B. (2019). Contrasting pathways for anaerobic methane oxidation in gulf of mexico cold seep sediments. *Appl. Environ. Sci.* 4:e00091-18. 10.1128/mSystems.00091-18 30834326 PMC6392090

[B196] Villar I. Alves D. Garrido J. Mato S. (2016). Evolution of microbial dynamics during the maturation phase of the composting of different types of waste. *Waste Manag.* 54:83. 10.1016/j.wasman.2016.05.011 27236404

[B197] Visser A. Gao Y. Lettinga G. (1993). Effects of pH on methanogenesis and sulfate reduction in thermophilic (55C) UASB reactors. *Bioresour. Technol.* 44 113–121. 10.1016/0960-8524(93)90184-d

[B198] Wagner D. Lipski A. Embacher A. Gattinger A. (2005). Methane fluxes in permafrost habitats of the Lena Delta: effects of microbial community structure and organic matter quality. *Environ. Microbiol.* 7 1582–1592. 10.1111/j.1462-2920.2005.00849.x 16156731

[B199] Wang J. Cai C. Li Y. Hua M. Wang J. Yang H. (2019). Denitrifying anaerobic methane oxidation: a previously overlooked methane sink in intertidal zone. *Environ. Sci. Technol.* 53 203–212. 10.1021/acs.est.8b05742 30457852

[B200] Wang J. Krause S. Muyzer G. Meima-Franke M. Laanbroek H. J. Bodelier P. L. E. (2012). Spatial patterns of iron- and methane-oxidizing bacterial communities in an irregularly flooded, riparian wetland. *Front. Microbiol.* 3:64. 10.3389/fmicb.2012.00064 22375139 PMC3284728

[B201] Wang Y. Tang W. Qiao J. Song L. (2015). Occurrence and prevalence of antibiotic resistance in landfill leachate. *Environ. Sci. Pollut. Res.* 22:12525. 10.1007/s11356-015-4514-7 25903180

[B202] Wheless E. Pierce J. (2004). “Siloxanes in landfill and digester gas update,” in *Proc. 27th. Annual SWANA LFG Symposium*, Los Angeles.

[B203] Wolfe R. S. (1979). “Methanogenesis,” in *Microbial Biochemistry, International Review of Biochemistry*, Vol. 21 ed. Quayle J. R. (Baltimore, MD: University Park Press).

[B204] Wu D. Chen G. Zhang X. Yang K. Xie B. (2017). Change in microbial community in landfill refuse contaminated with antibiotics facilitates denitrification more than the increase in ARG over long-term. *Nat. Sci. Rep.* 7:41230. 10.1038/srep41230 28120869 PMC5264584

[B205] Wu D. Huang Z. Yang K. Graham D. Xie B. (2015). Relationships between antibiotics and antibiotic resistance gene levels in municipal solid waste leachates in Shanghai, China. *Environ. Sci. Technol.* 49:4122. 10.1021/es506081z 25760223

[B206] Yadav S. Kundu S. Ghosh S. K. Maitra S. S. (2015). Molecular analysis of methanogen richness in landfill and marshland targeting 16S rDNA sequences. *Archaea* 2015 563414–563419. 10.1155/2015/563414 26568700 PMC4623359

[B207] Yan Z. Joshi P. Gorski C. A. Ferry J. G. (2018). A biochemical framework for anaerobic oxidation of methane driven by Fe(III)-dependent respiration. *Nat. Commun.* 9:1642. 10.1038/s41467-018-04097-9 PMC5915437 29691409

[B208] Yargicoglu E. N. Reddy K. R. (2017). Microbial abundance and activity in biochar-amended landfill cover soils: evidence from large-scale column and field experiments. *J. Environ. Eng.* 143:04017058. 10.1061/(asce)ee.1943-7870.0001254 29515898

[B209] Yesiller N. Hanson J. L. (2003). “Analysis of temperatures at a municipal solid waste landfill,” in *Sardinia 2003, Ninth International Waste Management and Landfill Symposium*, eds Christensen (Italy: CISA), 1–10.

[B210] Zahedi S. (2018). Energy efficiency: importance of indigenous microorganisms contained in the municipal solid wastes. *Waste Manag.* 78 763–769. 10.1016/j.wasman.2018.06.035 32559968

[B211] Zarasvand K. A. Rai V. R. (2013). Microorganisms: induction and inhibition of corrosion in metals. *Int. Biodeteriorat. Biodegradat.* 87 66–74. 10.1016/j.ibiod.2013.10.023

[B212] Zeng G. Liu L. Xue Q. Wan Y. Ma J. Zhao Y. (2017). Experimental study of the porosity and permeability of municipal solid waste. *Environ. Prog. Sustain. Energy* 36 1694–1699. 10.1002/ep.12632

[B213] Zhou C. Jiang D. Zhoa Z. (2017). Quantification of greenhouse gas emissions from the predisposal stage of municipal solid waste management. *Environ. Sci. Technol.* 51 320–327. 10.1021/acs.est.6b05180 27943673

[B214] Zhu S. Chan G. Y. S. Cai K.-L. Qu L.-H. Huang L.-N. (2007). Leachates from municipal solid waste disposal sites harbor similar, novel nitrogen-cycling bacterial communities. *FEMS Microbiol. Lett.* 267 236–242. 10.1111/j.1574-6968.2006.00560.x 17169002

[B215] Zyoud S. H. Al-Jabi S. W. Sweileh W. M. Al-Khalil S. Zyoud S. H. Sawalha A. F. (2015). The Arab world’s contribution to solid waste literature: a bibliometric analysis. *J. Occup. Med. Toxicol.* 10:35. 10.1186/s12995-015-0078-1 PMC4574093 26388930

